# MCPIP1 inhibits Wnt/β-catenin signaling pathway activity and modulates epithelial-mesenchymal transition during clear cell renal cell carcinoma progression by targeting miRNAs

**DOI:** 10.1038/s41388-021-02062-3

**Published:** 2021-10-16

**Authors:** Judyta Gorka, Paulina Marona, Oliwia Kwapisz, Agnieszka Waligórska, Ewelina Pospiech, Jurek W. Dobrucki, Janusz Rys, Jolanta Jura, Katarzyna Miekus

**Affiliations:** 1 Department of General Biochemistry, Faculty of Biochemistry, Biophysics and Biotechnology, Jagiellonian University, Gronostajowa 7, 30-387 Krakow, Poland; 2 Department of Cell Biophysics, Faculty of Biochemistry, Biophysics and Biotechnology, Jagiellonian University, Gronostajowa 7, 30-387 Krakow, Poland; 3 Human Genome Variation Research Group, Malopolska Centre of Biotechnology, Jagiellonian University, Gronostajowa 7A, 30-387 Krakow, Poland; 4 Department of Tumor Pathology, Centre of Oncology, Maria Skłodowska-Curie Memorial Institute, Cracow Branch, Garncarska 11, 31-115 Krakow, Poland

**Keywords:** Renal cell carcinoma, Cell signalling, Cytoskeleton, Mechanisms of disease, Cancer genetics

## Abstract

Epithelial-mesenchymal transition (EMT) refers to the acquisition of mesenchymal properties in cells participating in tumor progression. One hallmark of EMT is the increased level of active β-catenin, which can trigger the transcription of Wnt-specific genes responsible for the control of cell fate. We investigated how Monocyte Chemotactic Protein-1-Induced Protein-1 (MCPIP1), a negative regulator of inflammatory processes, affects EMT in a clear cell renal cell carcinoma (ccRCC) cell line, patient tumor tissues and a xenotransplant model. We showed that MCPIP1 degrades miRNAs via its RNase activity and thus protects the mRNA transcripts of negative regulators of the Wnt/β-catenin pathway from degradation, which in turn prevents EMT. Mechanistically, the loss of MCPIP1 RNase activity led to the upregulation of miRNA-519a-3p, miRNA-519b-3p, and miRNA-520c-3p, which inhibited the expression of Wnt pathway inhibitors (SFRP4, KREMEN1, CXXC4, CSNK1A1 and ZNFR3). Thus, the level of active nuclear β-catenin was increased, leading to increased levels of EMT inducers (SNAI1, SNAI2, ZEB1 and TWIST) and, consequently, decreased expression of E-cadherin, increased expression of mesenchymal markers, and acquisition of the mesenchymal phenotype. This study revealed that MCPIP1 may act as a tumor suppressor that prevents EMT by stabilizing Wnt inhibitors and decreasing the levels of active β-catenin and EMT inducers.

## Introduction

The Wnt signaling pathway is a key molecular cascade that regulates cell fate in animals. In the canonical Wnt pathway, β-catenin is the key effector responsible for signal transduction to the nucleus and the activation of Wnt-specific genes responsible for the control of cell fate [1]. When Wnt signaling is inactive, active β-catenin is recruited to a destruction complex, in which casein kinase 1 (CK1) phosphorylates β-catenin at serine 45 (S45), priming β-catenin for subsequent phosphorylation by glycogen synthase kinase 3 (GSK3). These phosphorylation events mark β-catenin for subsequent ubiquitination and proteasomal degradation and prevent β-catenin from translocating to the nucleus [2–4]. Wnt/β-catenin signaling is also inhibited by negative regulators of Wnt signaling. Decreased expression of negative regulators of Wnt signaling, including SFRP1, DKK-3, and CXXC4, is correlated with increased cytoplasmic β-catenin levels and tumor progression [5–7]. On the other hand, the phosphorylation of β-catenin by protein kinase B (AKT) and PKM1/2 helps to maintain its stability, reduces its interaction with E-cadherin, promotes Wnt signaling and enhances its transcriptional activity [8, 9]. Active β-catenin directly binds to transcription factors associated with the promoters of the key epithelial-mesenchymal transition (EMT) inducers SLUG, ZEB1, and TWIST and induces the expression of these inducers [10].

EMT is a key element of tumor progression that facilitates tumor cell migration and invasion, consequently supporting metastasis [11]. Inflammation is an important EMT inducer during cancer progression [12, 13]. Monocyte chemotactic protein-1-induced protein-1 (MCPIP1) regulates inflammation and degrades the mRNA transcripts of proinflammatory cytokines such as IL-6, IL-1β, IL-12 and IL-2 [14–16]. MCPIP1 physically interacts with stem-loop structures in the 3′ untranslated region (UTR) of transcripts through its PIN domain, causing mRNA destabilization followed by degradation [17]. MCPIP1 not only downregulates a set of mRNAs but also suppresses the production of mature miRNAs by cleaving the terminal loops of precursor miRNAs, thus counteracting Dicer1 activity [18, 19]. MCPIP1 is known to regulate the viability, proliferation, and apoptosis of tumor cells in the process of tumorigenesis [20–26]. Our group has already demonstrated that a low MCPIP1 protein level correlates with clear cell renal cell carcinoma (ccRCC) progression, increased tumor vascularization, and the metastatic process [24]. However, the mechanism by which MCPIP1 regulates EMT and tumor progression is unknown.

In the present study, we show for the first time that the MCPIP1 protein is crucial for regulating the expression of negative regulators of canonical Wnt signaling (SFRP4, KREMEN1, CXXC4, CSNK1A1 and ZNRF3), inhibiting the activation of β-catenin and maintaining the epithelial phenotype. We present evidence that the RNase activity of MCPIP1 might mediate tumor progression by controlling the transcriptional activation of β-catenin; promoting the expression of the transcription factors SNAI1, SNAI2, TWIST, and ZEB1; and inducing EMT.

## Results

### The RNase activity of MCPIP1 regulates the levels of mesenchymal markers

**Fig. 1:**
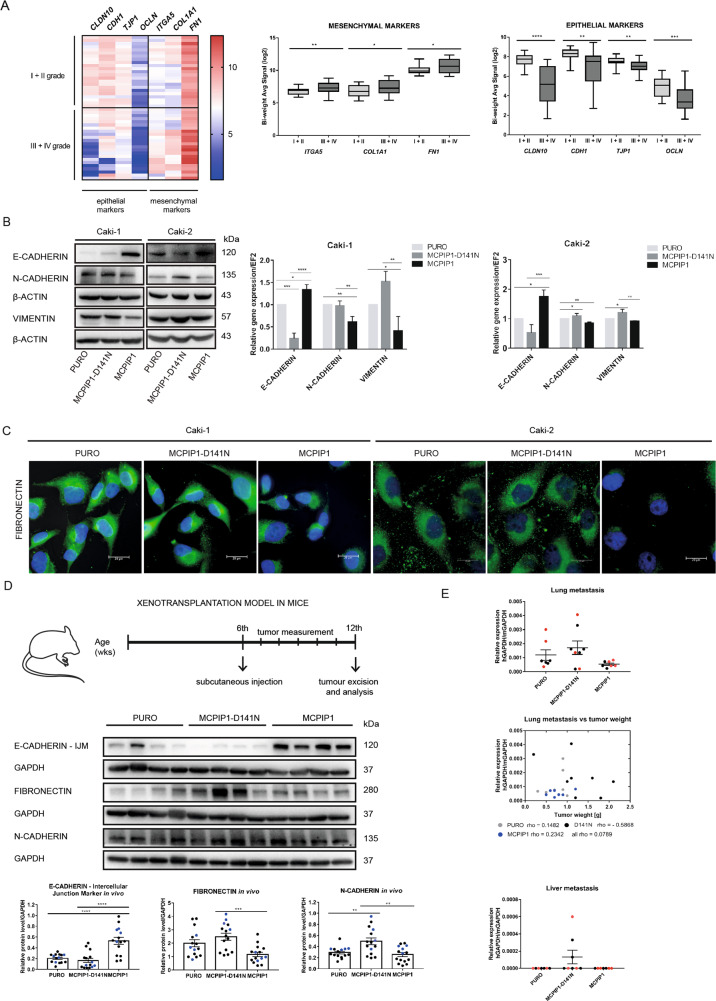
Influence of MCPIP1 on EMT markers. **A** Hierarchical clustering of 7 genes that are significantly associated with ccRCC tumor progression and epithelial-mesenchymal transition. Each row represents an individual tissue sample. The scale represents gene expression levels in log_2_ scale. On the right, quantification of the signal from microarray; *n* (I + II) = 23, *n* (III + IV) = 23. Statistics was performed using one-way ANOVA between subjects (unpaired). **B** Effect of MCPIP1 overexpression (MCPIP1) or mutation (D141N) on EMT markers. Left panel, representative western blot with β-actin as a loading control for the Caki-1 and Caki-2 cell line; right panel, mRNA levels of EMT markers in Caki-1 and Caki-2 cells, quantified with real-time PCR, EF2 was used as the reference gene. The results are presented as the mean ± SD of three independent experiments. *P* values were estimated using one-way ANOVA, *P < 0.05, **P < 0.01, ***P < 0.001, ****P < 0.0001. **C** Immunofluorescence staining of fibronectin in Caki-1 and Caki-2 cells after overexpression of MCPIP1 (MCPIP1), mutation of MCPIP1 (MCPIP1-D141N) and in control cells (PURO). DAPI for nuclei; fibronectin antibody labeled with fluorescent dye AlexaFluor 488, scale bar = 20 µm. **D** Effect of MCPIP1 overexpression and mutation on EMT markers in xenotransplantation model in mice. Tumors were collected 6 weeks after subcutaneous injection of Caki-1 cell line with MCPIP1 overexpression (MCPIP1), mutation (D141N) and control (PURO). Representative western blot analysis of EMT markers in tumors with overexpression, mutation of MCPIP1 and control (PURO). Middle panel, densitometric quantification of protein levels in tumors. GAPDH as a loading control. Animal studies involved 45 NOD-SCID mice: PURO *N* = 15, MCPIP1-D141N *N* = 15, MCPIP1 *N* = 15. The results are presented as means ± SD. *P* values were estimated using one-way ANOVA, *P < 0.05, **P < 0.01, ***P < 0.001. **E** mRNA analysis of lung and liver metastasis using real-time PCR in NOD-SCID mice after 6 weeks of subcutaneous injection with Caki-1 GFP with MCPIP1 overexpression (MCPIP1), mutation (D141N) and control. The study involved 8 NOD-SCID mice: PURO *N* = 7, MCPIP1-D141N *N* = 8, MCPIP1 *N* = 7. *Gapdh* was used as the reference gene. The results are presented as means ± SEM. *P* values were estimated using one-way ANOVA. Below, graph showing the dependence of the lung metastasis and tumor weight after 6 weeks of subcutaneous injection of Caki-1 cell line with MCPIP1 overexpression (MCPIP1), mutation (D141) or control (PURO); coefficient of correlation (rho) of Spearman Correlation test is shown as well.

We previously showed that the level of MCPIP1 negatively correlates with the progression of ccRCC [22, 24]. In this study, we verified the levels of key transcripts in the EMT process in samples from patients with all grades (I–IV) of ccRCC (Fig. 1A). We observed increased levels of the mesenchymal markers *ITGA5*, *COL1A1* and *FN1* and decreased levels of *CLDN10*, *CDH1*, *TJP1* and *OCLN*, which are characteristic of the epithelial phenotype, in ccRCC patients versus healthy volunteers (Fig. 1A). We evaluated the effects of the overexpression of MCPIP1 or MCPIP1 with the D141 point mutation (MCPIP1-D141), which completely abolishes MCPIP1 RNase activity [27], on the levels of EMT markers. Upregulation of MCPIP1 decreased the levels of N-cadherin and vimentin, characteristic markers of the mesenchymal phenotype. Moreover, we observed a significant increase in the transcript and protein levels of the main epithelial marker E-cadherin (Fig. 1B; Supplementary Fig. S1A, B, D). Interestingly, mutation of the RNase domain of MCPIP1 (MCPIP1-D141N) resulted in high protein and mRNA levels of N-cadherin and vimentin and a low level of E-cadherin (Fig. 1B; Supplementary Fig. S1B, D). Immunofluorescence staining confirmed increased accumulation of fibronectin in cells lacking MCPIP1 RNase activity (Fig. 1C, Supplementary Fig. S1C). We expanded our in vitro model by assessing cells with stable MCPIP1 knockdown at the mRNA and protein levels (shMCPIP1 cells). We found that MCPIP1 depletion in ccRCC cells significantly enhanced mesenchymal marker expression (Supplementary Fig. S1E). To confirm that MCPIP1 protects against the acquisition of the mesenchymal phenotype, we performed a study in Foxn1^nu^/Foxn1^nu^ and NOD-SCID mice subcutaneously injected with ccRCC cells overexpressing the MCPIP1 protein (MCPIP1) or its RNase-depleted form (MCPIP1-D141) or silencing MCPIP1 (shMCPIP1) (Fig. 1D, Supplementary Fig. S1F). Tumors harboring mutations in the RNase domain of MCPIP1 or with downregulation of MCPIP1 were characterized by high levels of mesenchymal markers (N-cadherin and fibronectin), whereas MCPIP1 overexpression resulted in high levels of epithelial E-cadherin (Fig. 1D, Supplementary Fig. S1F). Mesenchymal features are responsible for the invasive phenotype and metastatic behavior of many tumor types [28]. Our studies showed that the lack of MCPIP1 RNase activity increased the metastatic ability in vivo (Fig. 1E). We observed that mice subcutaneously injected with Caki-1 cells carrying a D141 mutation had more lung and liver metastases than did mice injected with control cells (Fig. 1E). Stable upregulation of MCPIP1 resulted in a decrease in the number of metastatic tumor cells (Fig. 1E). We observed that the inhibition of MCPIP1 affected tumor growth, increased the levels of N-cadherin and fibronectin, and reduced the level of E-cadherin (Supplementary Fig. S1F).

### MCPIP1 prevents EMT in normal RPTEC/TERT1 cells

**Fig. 2:**
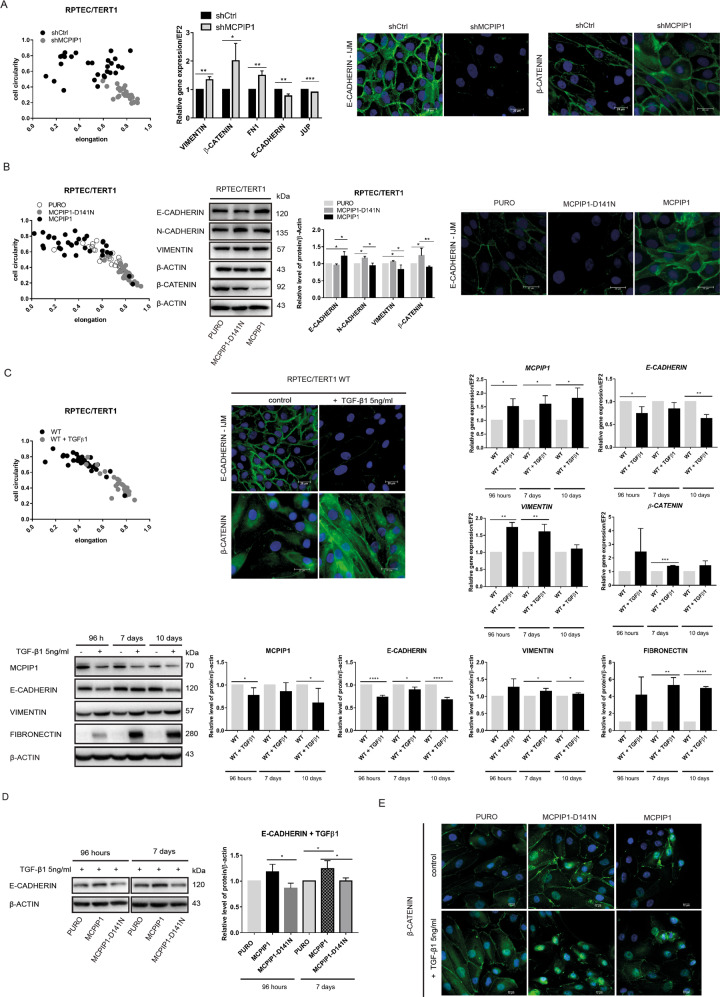
Effect of the MCPIP1 level in normal RPTEC/TERT1 cells on EMT. **A** Effect of MCPIP1 downregulation (shMCPIP1) in RPTEC/TERT1 cell line. Morphological analysis shows the relationship between cell circularity and elongation (left panel). Effect of MCPIP1 downregulation on mRNA expression of E-*cadherin*, *JUP*, *Vimentin*, *β-catenin*, and *FN1* in RPTEC/TERT1 cell line (middle panel). The results are presented as the mean ± SD of three independent experiments. *P* values were estimated using two-tailed unpaired Student’s *t* test, *p < 0.05, **p < 0.01, ***p < 0.001. Right panel, immunofluorescence staining to visualize cell morphology by E-cadherin – IJM and β-catenin in RPTEC/TERT1 cells after downregulation of MCPIP1 (shMCPIP1). DAPI for nuclei; E-cadherin - IJM antibody labeled with fluorescent dye AlexaFluor 488; β-catenin antibody labeled with fluorescent dye AlexaFluor 546, scale bar = 20 µm. **B** Effect of MCPIP1 overexpression (MCPIP1) or mutation (MCPIP1-D141N) in RPTEC/TERT1 cell line. Morphological analysis shows the relationship between cell circularity and elongation (left panel). Western blot analysis of EMT markers in RPTEC/TERT1 cells: E-cadherin, N-cadherin, Vimentin, and β-catenin after overexpression of MCPIP1, MCPIP1-D141N and PURO (middle panel). Representative western blot with β-actin as a loading control. Right panel, immunofluorescence staining to visualize cell morphology by E-cadherin – IJM in RPTEC/TERT1 cells after overexpression of MCPIP1, MCPIP1-D141N and PURO. DAPI for nuclei; scale bar = 20 µm. **C** Morphological analysis shows the relationship between cell circularity and elongation (left panel) after 24 h TGF-β1 stimulation in RPTEC/TERT1 cells. Staining for E-cadherin – IJM, main epithelial marker after 24 h TGF-β1 stimulation in RPTEC/TERT1 cells. DAPI for nuclei; scale bar = 20 µm. On the right, mRNA expression of MCPIP1, E-cadherin, Vimentin, and β-catenin after TGF-β1 stimulation in RPTEC/TERT1 cells at various time points: 96 h and 7, 10 days. *EF2* was used as the reference gene. Below, western blot and densitometric quantification showing the level of MCPIP1, E-cadherin, Vimentin, and fibronectin after TGF-β1 stimulation in RPTEC/TERT1 cells. The results are presented as the mean ± SD of three independent experiments. *P* values were estimated using one-way ANOVA, *P < 0.05, **P < 0.01, ***P < 0.001. **D** Western blot analysis and densitometric quantification of the level of E-cadherin in RPTEC/TERT1 after overexpression of MCPIP1 (MCPIP1), mutation (MCPIP1-D141N) and in control cells (PURO) stimulated with TGF-β1 after 96 h and 7 days. The results are presented as the mean ± SD of three independent experiments. *P* values were estimated using one-way ANOVA, *P < 0.05. **E** Immunofluorescence staining of fibronectin in RPTEC/TERT1 cell line after overexpression of MCPIP1, MCPIP1-D141N and PURO. DAPI for nuclei; fibronectin antibody labeled with fluorescent dye AlexaFluor 546, scale bar = 10 µm.

The results regarding the impact of MCPIP1 on the regulation of the EMT process directed our interest toward the role of MCPIP1 in normal cells. As a model, we used an immortalized cell line derived from renal proximal tubule epithelial cells (RPTECs), which did not exhibit chromosomal abnormalities [29]. Silencing of MCPIP1 in normal epithelial kidney cells induced a cell morphological change to a more elongated shape; increased the expression of the mesenchymal markers vimentin, β-catenin, and fibronectin; and decreased the expression of E-cadherin and JUP (Fig. 2A, Supplementary Fig. S2A). The loss of MCPIP1 RNase activity (MCPIP1-D141N) in RPTEC/TERT1 cells induced the acquisition of mesenchymal cell morphology and increased the levels of the mesenchymal markers N-cadherin and vimentin (Fig. 2B, Supplementary Fig. S2B). Overexpression of MCPIP1 significantly decreased N-cadherin, vimentin and β-catenin levels and increased E-cadherin levels (Fig. 2B, Supplementary Fig. S2B). The major activator of EMT is TGFβ. We sought to determine whether the level of MCPIP1 changes in normal cells after the activation of EMT by TGFβ1. TGFβ1 stimulation induced morphological changes in RPTECs, including characteristic elongation, anteroposterior polarity, the presence of a leading edge, and limited intercellular connections (Fig. 2C, Supplementary Fig. S2C). As expected, the transcript and protein levels of E-cadherin decreased, whereas the levels of vimentin and fibronectin increased (Fig. 2C). Interestingly, the protein level of MCPIP1 also systematically decreased (Fig. 2C). Interestingly, overexpression of MCPIP1 in RPTECs protected these cells against the effects of TGF-β1 stimulation. MCPIP1-overexpressing RPTECs had an unchanged or even increased level of the main epithelial marker E-cadherin, whereas cells lacking MCPIP1 RNase activity (MCPIP1-D141N) were not protected and exhibited a significantly reduced E-cadherin level after stimulation with TGF-β1 (Fig. 2D). Moreover, TGF-β1 stimulation increased the level of β-catenin in cells lacking MCPIP1 RNase activity, whereas MCPIP1 overexpression protected against the increase in β-catenin (Fig. 2E).

### The RNase activity of MCPIP1 regulates the expression level and localization of β-catenin

**Fig. 3:**
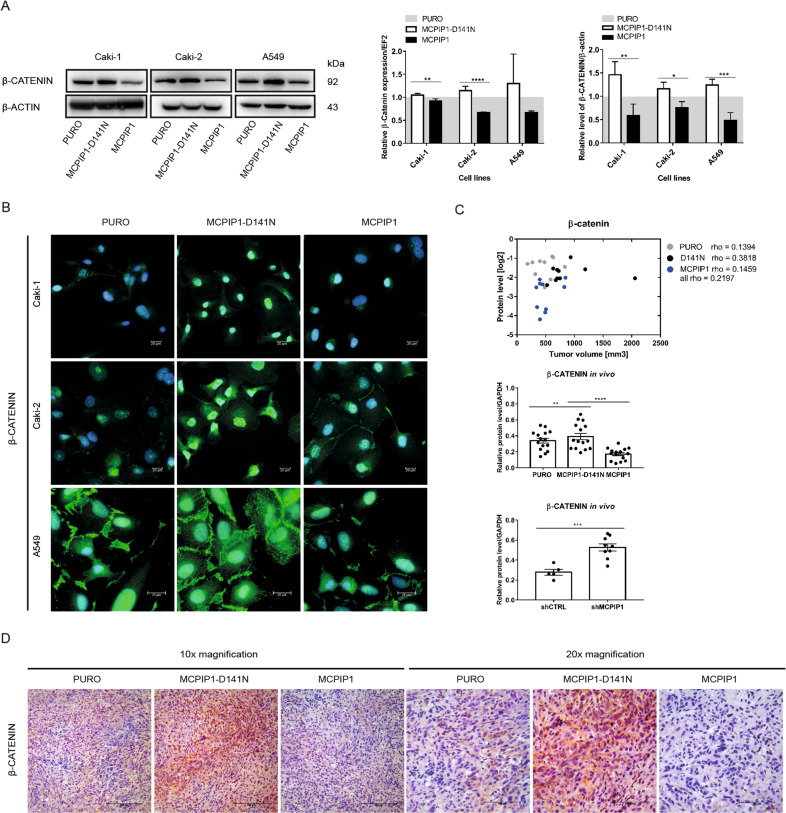
Influence of MCPIP1 on β-catenin. **A** Representative western blot of β-catenin from ccRCC and A549 cell lines with β-actin as the loading control and densitometric quantification. mRNA expression of β-catenin in cell lines, quantified with real-time PCR, *EF2* was used as the reference gene. The results are presented as the mean ± SD of three independent experiments. *P* values were estimated using one-way ANOVA, *P < 0.05, **P < 0.01, ***P < 0.001. **B** Immunofluorescence staining on β-catenin in Caki-1, Caki-2 and A549 cells after overexpression of MCPIP1 (MCPIP1) and mutation (MCPIP1-D141N) with control (PURO). DAPI for nuclei; β-catenin antibody labeled with fluorescent dye AlexaFluor 546, scale bar = 20 µm. **C** Effect of MCPIP1 overexpression, mutation and downregulation on β-catenin in xenotransplantation model in mice. Graph showing the dependence of the level of β-catenin and tumor volume after 6 weeks of subcutaneous injection of Caki-1 cell line with MCPIP1 overexpression (MCPIP1), mutation (D141) or control (PURO); coefficient of correlation (rho) of Spearman Correlation test is shown as well. Below densitometric quantification of β-catenin in tumors. GAPDH as the loading control. Tumors were collected 6 weeks after subcutaneous injection of Caki-1 cell line with MCPIP1 overexpression (MCPIP1), mutation (D141) or downregulation (shMCPIP1). Animal studies involved 45 NOD-SCID mice: PURO *N* = 15, MCPIP1-D141N *N* = 15, MCPIP1 *N* = 15 and 9 Foxn1^nu^/Foxn1^nu^ mice: shCTRL *N* = 5, shMCPIP1 *N* = 9. The results are presented as means ± SD. *P* values were estimated using one-way ANOVA and two-tailed unpaired Student’s *t* test, *P < 0.05, **P < 0.01, ***P < 0.001, ****P < 0.0001. **D** β-catenin IHC staining of tumor sections. Immunohistochemical evaluation was performed using primary β-catenin antibody and EnVision Detection Systems Peroxidase/DAB ((3,30-Diaminobenzidine) Rabbit/Mouse (Dako), scale bar, 100 and 200 µm.

One hallmark of EMT is the loss of E-cadherin function, which results in the dissociation of β-catenin from the membrane and an increase in β-catenin-dependent transcription [30, 31]. Therefore, in further experiments, we assessed the influence of MCPIP1 expression on the level of β-catenin. MCPIP1-overexpressing cells had significantly lower protein and mRNA levels of β-catenin than control cells (Fig. 3A). Even more interestingly, loss of MCPIP1 RNase activity led to visible translocation of β-catenin into the nucleus, as determined by immunofluorescence staining (Fig. 3B). We correlated the β-catenin level with the size of tumors overexpressing MCPIP1 or lacking MCPIP1 RNase activity (D141N) generated in mice. Loss of MCPIP1 RNase activity led to increases in the β-catenin level and tumor size (Fig. 3C). Tumors lacking RNase activity had a significantly higher level of β-catenin than control or MCPIP1-overexpressing tumors in the two investigated mouse models (Fig. 3C). As expected, silencing MCPIP1 resulted in an increase in the β-catenin level (Fig. 3C). Immunohistochemical staining of β-catenin showed increased levels in tumors lacking MCPIP1 RNase activity and weak signals in MCPIP1-overexpressing tumors (Fig. 3D).

### MCPIP1 regulates the activity of β-catenin in ccRCC cells

**Fig. 4:**
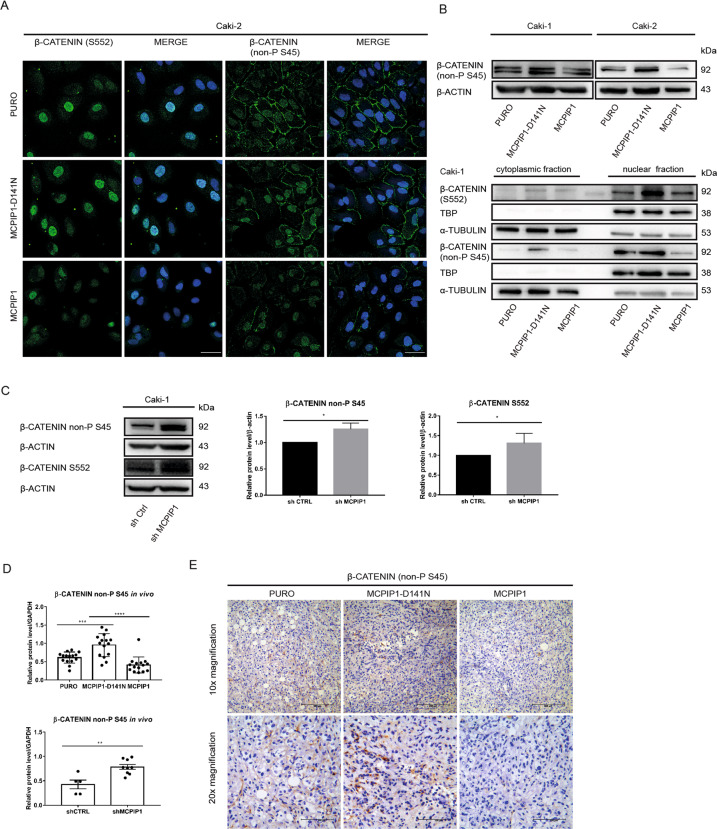
MCPIP1 affects the β-catenin activity. **A** Confocal analysis of the nuclear location of phospho-β-catenin (S552) and active β-catenin in the Caki-2 cell line with MCPIP1 overexpression (MCPIP1), the mutated form of MCPIP1 (MCPIP1-D141N) and control cells (PURO). DAPI for nuclei; phospho-β-catenin (S552) antibody and anti-non-phospho (Active) β-Catenin antibody labeled with fluorescent dye AlexaFluor 488, scale bar = 20 µm. **B** Western blot analysis of active (non-P S45) β-catenin from ccRCC cell line from total lysate with β-actin as a loading control. Below, representative western blot of phospho β-catenin (S552) and active (non-P S45) β-catenin from Caki-1 divided to cytoplasmic and nuclear fraction with TBP as a loading control for nuclear fraction and α-tubulin as a loading control for cytoplasmic fraction. **C** Western blot analysis and densitometric quantification of the level of phospho β-catenin (S552) and active (non-P S45) β-catenin from Caki-1 after downregulation of MCPIP1 (shMCPIP1) and control (shCtrl) with β-actin as the loading control. The results are presented as the mean ± SD of three independent experiments. *P* values were estimated using two-tailed unpaired Student’s *t* test, *P < 0.05. **D** Effect of MCPIP1 overexpression, mutation, and downregulation on active (non-P S45) β-catenin in xenotransplantation model in mice. Densitometric quantification of active (non-P S45) β-catenin in tumors. GAPDH as the loading control. Tumors were collected 6 weeks after subcutaneous injection of Caki-1 cell line with MCPIP1 overexpression (MCPIP1), mutation (D141) or downregulation (shMCPIP1) with control (PURO) and (shCTRL). Animal studies involved 45 NOD-SCID mice: PURO *N* = 15, MCPIP1-D141N *N* = 15, MCPIP1 *N* = 15 and 9 Foxn1^nu^/Foxn1^nu^ mice: shCTRL *N* = 5, shMCPIP1 *N* = 9. The results are presented as means ± SD. *P* values were estimated using one-way ANOVA and two-tailed unpaired Student’s *t* test, *P < 0.05, **P < 0.01, ***P < 0.001, ****P < 0.0001. **E** Active (non-P S45) β-catenin IHC staining of tumor sections. Immunohistochemical evaluation was performed using primary active (non-P S45) β-catenin antibody and EnVision Detection Systems Peroxidase/DAB ((3,30-Diaminobenzidine) Rabbit/Mouse (Dako), scale bar, 100 and 200 µm.

The regulation of β-catenin occurs via its phosphorylation, which is crucial for its activity and degradation. β-Catenin can be phosphorylated on Ser45 and then on Ser33, Ser37, and Thr41, which mediates its degradation by the proteasome [4]. On the other hand, phosphorylation of Ser552 by AKT stabilizes β-catenin and increases its transcriptional activity [8, 9]. Immunofluorescence staining and western blot analysis of the total pool of active β-catenin (not phosphorylated on Ser45; non-P S45) showed the strongest signal in cells expressing MCPIP1 with the PIN domain mutation that abolished its RNase function (MCPIP1-D141N). Active β-catenin (non-P S45) was localized both in the nucleus and near the cell membrane (Fig. 4A, B; Supplementary Fig. S3A). The RNase activity of MCPIP1 also regulates the level of transcriptionally active (S552) β-catenin. The strongest signal representing stabilized β-catenin (S552) was observed in the nucleus of cells lacking MCPIP1 RNase activity (Fig. 4B, Supplementary Fig. S3A). Overexpression of MCPIP1 significantly reduced the levels of both active forms of β-catenin (Fig. 4A, B; Supplementary Fig. S3A). Silencing of MCPIP1 in ccRCC cells increased the levels of active β-catenin (non-P S45) and transcriptionally active (S552) forms of β-catenin (Fig. 4C).

Moreover, tumors lacking MCPIP1 RNase activity had the highest level of active β-catenin (non-P S45) in the two investigated mouse models (Fig. 4D). Tumors with downregulation of MCPIP1 (shMCPIP1) also exhibited a significant increase in the active form of β-catenin (non-P S45) (Fig. 4D). In contrast, overexpression of MCPIP1 in tumors resulted in a decrease in the level of active β-catenin (Fig. 4D). Immunohistochemical staining revealed the strongest signal of active β-catenin (non-P S45) in tumors harboring the mutation in MCPIP1 that abolished its RNase activity (Fig. 4E).

### MCPIP1 regulates the expression of negative regulators of the Wnt pathway

**Fig. 5:**
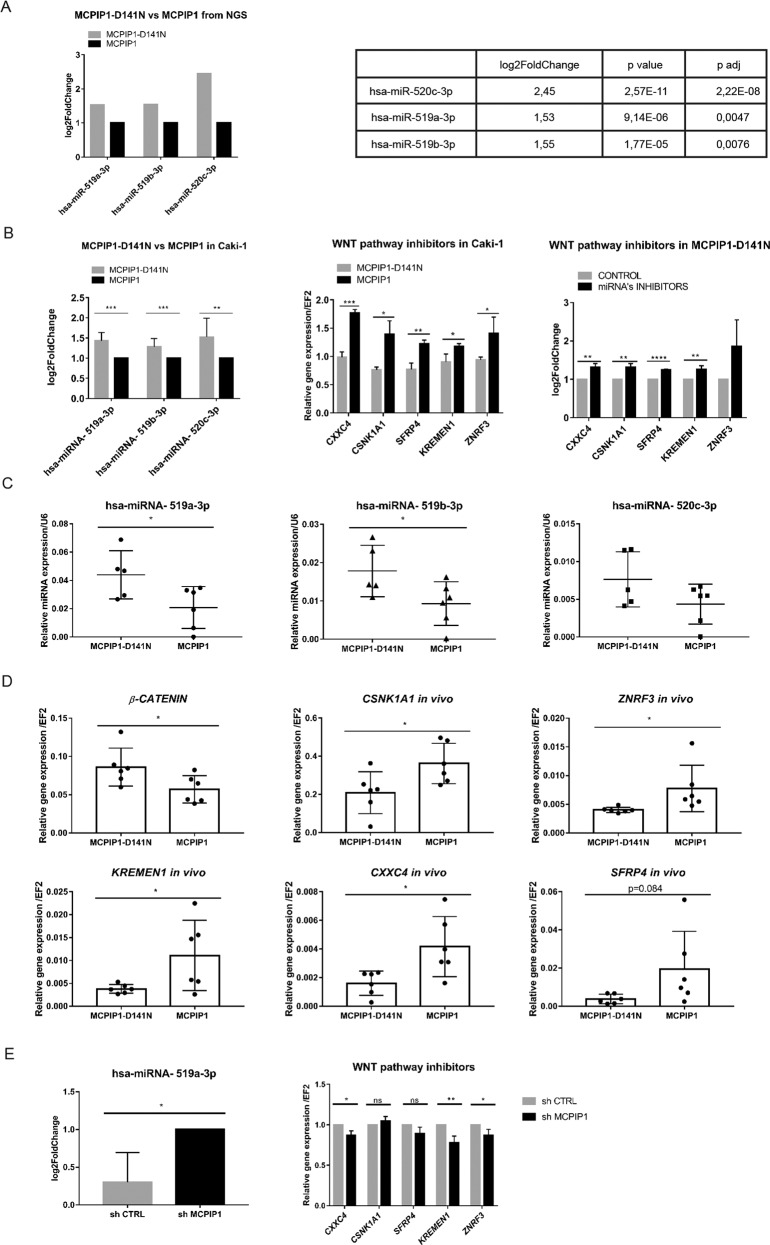
Influence of MCPIP1 on negative regulators of the Wnt pathway. **A** The Next-Generation Sequencing results of miRNA-519a-3p, miRNA-519b-3p, and miRNA-520c-3p in the Caki-1 cells overexpressing MCPIP1 and MCPIP1-D141N. *P* values for differentially expressed miRNAs were corrected for multiple comparisons using Benjamini–Hochberg approach and the results with the corrected *P* values < 0.05 were considered significant. **B** Analysis of miRNA’s level using real-time PCR method was performed in the Caki-1 cell line with overexpression of MCPIP1 and mutation of MCPIP1 (MCPIP1-D141N). *U6* was used as the reference gene. The results are presented as the mean ± SD of three independent experiments. *P* values were estimated using two-tailed unpaired Student’s *t* test, **P < 0.01, ***P < 0.001. Middle graph, analysis of mRNA level for inhibitors of the Wnt pathway: *SRFP4*, *KREMEN1*, *ZNRF3*, *CSNK1A1* and *CXXC4* in the Caki-1 cell line with overexpression of MCPIP1 (MCPIP1) and mutation (MCPIP1-D141N) using real-time PCR. *EF2* was used as the reference gene. The results are presented as the mean ± SD of three independent experiments. Right graph presents the influence of miRNA’s inhibitors. Analysis of mRNA levels of genes: *SRFP4*, *KREMEN1*, *ZNRF3*, *CSNK1A1* and *CXXC4* using real-time PCR. The experiment was performed on Caki-1 cells with the D141N mutation after treatment with miRNA’s inhibitors relative to control inhibitors. 24 h after seeding, to induce overexpression, doxycycline was added. After another 24 h, miRNA Power Inhibitors targeting miRNA-519a-3p, miRNA-519b-3p and miRNA-520c-3p were added at a total concentration of 3 µM (each 1 µM). Negative Control A was used at a concentration of 3 µM. *EF2* was used as the reference gene. The results are presented as the mean ± SD of three independent experiments. *P* values were estimated using two-tailed unpaired Student’s *t* test, *P < 0.05, **P < 0.01, ***P < 0.001, ****P < 0.0001. **C** Expression level of miRNA-519a-3p, miRNA-519b-3p, and miRNA-520c-3p in a xenotransplantation model in NOD-SCID mice with overexpression (MCPIP1) and mutation of MCPIP1 (MCPIP1-D141N). MCPIP1-D141N *N* = 5 mice, MCPIP1 *N* = 6. *U6* was used as the reference gene. The results are presented as means ± SD. *P* values were estimated using two-tailed unpaired Student’s *t* test, *P < 0.05. **D** Expression level of inhibitors of the Wnt pathway: *SRFP4*, *KREMEN1*, *ZNRF3*, *CSNK1A1* and *CXXC4* in a xenotransplantation model in NOD-SCID mice with overexpression (MCPIP1) and mutation of MCPIP1 (MCPIP1-D141N). MCPIP1-D141N *N* = 6 mice, MCPIP1 *N* = 6. *EF2* was used as the reference gene. The results are presented as means ± SD. *P* values were estimated using two-tailed unpaired Student’s *t* test, *P < 0.05. **E** Analysis of miRNA-519a-3p level using real-time PCR method was performed in the Caki-1 cell line with downregulation of MCPIP1 (shMCPIP1). *U6* was used as the reference gene. Right panel, expression level of inhibitors of the Wnt pathway: *SRFP4*, *KREMEN1*, *ZNRF3*, *CSNK1A1* and *CXXC4* in the Caki-1 cell line with downregulation of MCPIP1. *EF2* was used as the reference gene. The results are presented as the mean ± SD of three independent experiments. *P* values were estimated using two-tailed unpaired Student’s *t* test, *P < 0.05, **P < 0.01.

MCPIP1 has already been shown to directly regulate the biosynthesis of some miRNAs via its RNase activity [18, 19]. Reports also indicate that alterations in miRNA regulatory networks are critical for the induction of EMT in ccRCC [32]. To identify the potential mechanism by which MCPIP1 affects the EMT program, we examined the levels of miRNAs in cells overexpressing MCPIP1 or expressing the mutant form of MCPIP1 (D141N) using next-generation sequencing (NGS). We identified several miRNAs dependent on the RNase activity of MCPIP1; specifically, the levels of miRNA-519a-3p, miRNA-519b-3p, and miRNA-520c-3p were higher in cells lacking MCPIP1 RNase activity than in cells overexpressing MCPIP1 (Fig. 5A). To confirm the NGS results, we analyzed the levels of selected miRNAs by real-time RT-PCR. Loss of MCPIP1 RNase activity significantly increased the levels of miRNA-519a-3p, miRNA-519b-3p, and miRNA-520c-3p (Fig. 5B, left panel). We used the Diana microT-CDS database to screen target genes regulated by selected miRNAs and found that miRNA-519a-3p, miRNA-519b-3p, and miRNA-520c-3p inhibit the mRNA production or translation process of several genes, including SFRP4, ZNRF3, KREMEN1, CXXC4 and CSNK1A1, which act as negative regulators of Wnt signaling and reduce the level of active β-catenin (Supplementary Fig. S4). In addition, compared with the expression of the mutant form of MCPIP1, overexpression of MCPIP1 significantly increased the levels of these inhibitors (Fig. 5B, middle panel). Exposure of Caki-1 cells harboring mutations in the RNase domain of MCPIP1 to inhibitors of miRNA-519a-3p, miRNA-519b-3p, and miRNA-520c-3p led to increases in the levels of Wnt pathway inhibitors, confirming the importance of the RNase function of MCPIP1 in regulating the canonical Wnt pathway and the β-catenin level (Fig. 5B, right panel). In addition, we evaluated the influence of MCPIP1 on the expression of genes associated with the Wnt signaling pathway. We identified increases in the expression levels of 20 genes that inhibit Wnt pathway activity in cells overexpressing MCPIP1 compared with cells lacking MCPIP1 RNase activity (Supplementary Fig. S5). We then evaluated the levels of miRNAs dependent on MCPIP1 RNase activity in the NOD-SCID mouse model and found that tumors lacking MCPIP1 RNase activity exhibited increased levels of miRNA-519a-3p, miRNA-519b-3p, and miRNA-520c-3p compared to those in tumors overexpressing MCPIP1 (Fig. 5C). Our findings also showed that in the investigated xenograft model, overexpression of MCPIP1 led to higher mRNA levels of the Wnt pathway inhibitors *SFRP4*, *ZNRF3*, *KREMEN1*, *CXXC4*, and *CSNK1A1* than overexpression of MCPIP1 lacking RNase activity (Fig. 5D). Moreover, the mRNA level of β-catenin was inversely correlated with the levels of inhibitors of the Wnt pathway in tumors overexpressing MCPIP1 (Fig. 5D). We noticed that the downregulation of MCPIP1 in ccRCC cells significantly increased the level of miRNA-519a-3p and decreased the mRNA levels of the Wnt pathway inhibitors *CXXC4*, *KREMEN1*, and *ZNRF3* (Fig. 5E).

### The RNase activity of MCPIP1 regulates the level of transcription factors controlling EMT

**Fig. 6:**
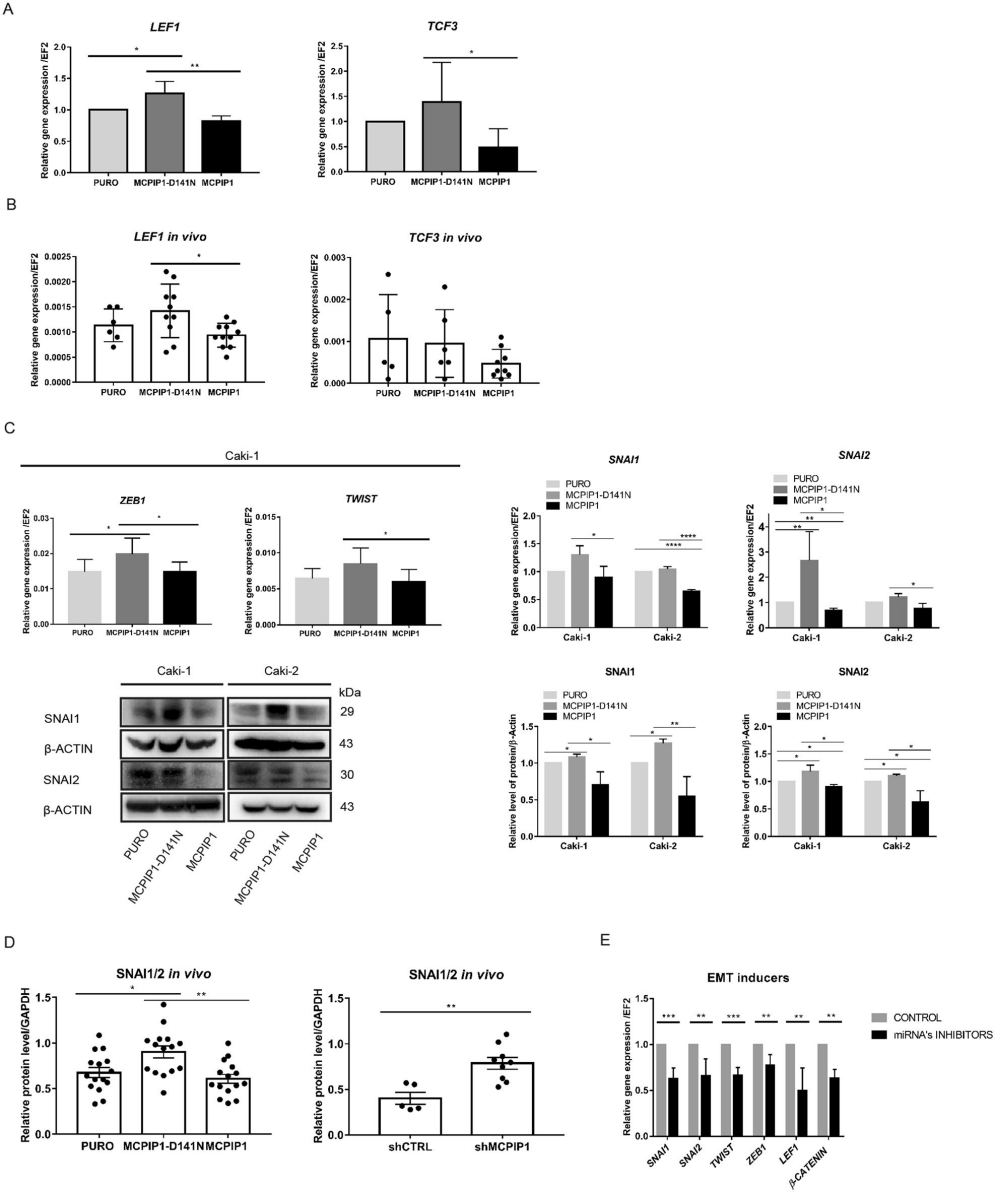
The RNase activity of MCPIP1 affects EMT inducers. **A** Analysis of mRNA level of *LEF1* and *TCF3* in the Caki-1 cell line after overexpression of MCPIP1 (MCPIP1), mutation (MCPIP1-D141N) and control (PURO). The results are presented as the mean ± SD of three independent experiments. *P* values were estimated using one-way ANOVA, *P < 0.05, **P < 0.01, ***P < 0.001, ****P < 0.0001. **B** Effect of MCPIP1 overexpression, mutation, and control on *LEF1* and *TCF3* in xenotransplantation model in mice. Tumors were collected 6 weeks after subcutaneous injection of Caki-1 cell line with MCPIP1 overexpression (MCPIP1) and mutation (D141N). Analysis of mRNA level of *LEF1* and *TCF3* in tumors. Animal studies involved 45 NOD-SCID mice of LEF1 for 27 mice a specific product was obtained in the real-time PCR reaction: PURO *N* = 6, MCPIP1-D141N *N* = 10, MCPIP1 *N* = 11. Animal studies involved 45 NOD-SCID mice of TCF3, for 20 mice a specific product was obtained in the real-time PCR reaction: PURO *N* = 5, MCPIP1-D141N *N* = 6, MCPIP1 *N* = 9. *EF2* was used as the reference gene. The results are presented as means ± SD. *P* values were estimated using one-way ANOVA, *P < 0.05, **P < 0.01, ***P < 0.001. **C** Analysis of mRNA level of *ZEB1* and *TWIST* in the Caki-1 cell line after overexpression of MCPIP1 (MCPIP1), mutation (MCPIP1-D141N) and control (PURO). On the right, analysis of mRNA level of *SNAI1* and *SNAI2* in both ccRCC cell lines. *EF2* was used as the reference gene. Below representative western blot of SNAI1 and SNAI2 in Caki-1 and Caki-2 cell lines with overexpression mutation and control with β-actin as the loading control. On the right, densitometric quantification, PURO was set to 1. The results are presented as the mean ± SD of three independent experiments. *P* values were estimated using one-way ANOVA, *P < 0.05, **P < 0.01, ***P < 0.001, ****P < 0.0001. **D** Effect of MCPIP1 overexpression, mutation and downregulation on EMT regulators in xenotransplantation model in mice. Tumors were collected 6 weeks after subcutaneous injection of Caki-1 cell line with MCPIP1 overexpression (MCPIP1), mutation (D141) or downregulation (shMCPIP1) with control (PURO) and (shCTRL). Densitometric quantification of protein level of SNAI1/2 with GAPDH as the loading control. Animal studies involved 45 NOD-SCID mice: PURO *N* = 15, MCPIP1-D141N *N* = 15, MCPIP1 *N* = 15 and 9 Foxn1^nu^/Foxn1^nu^ mice: shCTRL *N* = 5, shMCPIP1 *N* = 9. The results are presented as means ± SD. *P* values were estimated using one-way ANOVA and two-tailed unpaired Student’s *t* test, *P < 0.05, **P < 0.01. **E** Analysis the influence of miRNA’s inhibitors on EMT inducers. Analysis of mRNA levels of genes: *SNAI1*, *SNAI2*, *TWIST*, *ZEB1*, *LEF1* and *β-catenin* using real-time PCR. The experiment was performed on Caki-1 cells with the D141N mutation after treatment with miRNA’s inhibitors relative to control inhibitors. 24 h after seeding, to induce overexpression, doxycycline was added. After another 24 h, miRNA Power Inhibitors targeting miRNA-519a-3p, miRNA-519b-3p and miRNA-520c-3p were added at a total concentration of 3 µM (each 1 µM). Negative Control A was used at a concentration of 3 µM. *EF2* was used as the reference gene. The results are presented as the mean ± SD of three independent experiments. *P* values were estimated using two-tailed unpaired Student’s *t* test, *P < 0.05, **P < 0.01, ***P < 0.001, ****P < 0.0001.

One signaling pathway controlling EMT is the activated Wnt/β-catenin pathway. Active β-catenin associates with transcription factors of the TCF/LEF family and drives the transcription of Wnt/β‐catenin target genes. It has been previously shown that WNT/β-catenin signaling dysregulation is associated with renal cell carcinoma (RCC) carcinogenesis and progression [33]. We noticed that the lack of MCPIP1 RNase activity in Caki-1 cells significantly increased the expression of transcription factors of the Wnt pathway *LEF1* and *TCF3* (Fig. 6A). Moreover, we observed that the RNase activity of MCPIP1 regulates its expression in tumors isolated from nude mice injected with ccRCC cells (Fig. 6B). It has been demonstrated that the direct and indirect targets of β-catenin include EMT-induced transcription factors such as TWIST, ZEB1, SNAI1, and SNAI2 [1]. As the RNase activity of MCPIP1 regulates the level and activation of β-catenin, we assessed the influence of MCPIP1 overexpression or PIN domain mutation on the levels of EMT inducers. Loss of MCPIP1 RNase activity led to significant increases in both the mRNA and protein levels of TWIST, ZEB1, SNAI1, and SNAI2, while overexpression of MCPIP1 significantly reduced their expression (Fig. 6C). In addition, downregulation of MCPIP1 led to increased mRNA levels of SNAI1 and ZEB1 in normal kidney cells (Fig. S6A). The RNase activity of MCPIP1 was also crucial for maintaining the high levels of transcription factors responsible for the EMT process in the Foxn1^nu^/Foxn1^nu^ and NOD-SCID mouse xenotransplantation models (Fig. 6D). A similar effect was observed in mice bearing tumors with downregulation of MCPIP1 (shMCPIP1) (Fig. 6D).

Our studies also showed that exposure of Caki-1 cells harboring mutations in the RNase domain of MCPIP1 to inhibitors of miRNA-519a-3p, miRNA-519b-3p, and miRNA-520c-3p led to decreases in the levels of Wnt pathway inhibitors and increases in the expression of EMT inducers, confirming the importance of MCPIP1 in regulating the expression of transcription factors activating EMT (Fig. 6E).

### Wnt pathway inhibitors are downregulated during ccRCC progression with increases in the levels of β-catenin and EMT inducers

**Fig. 7:**
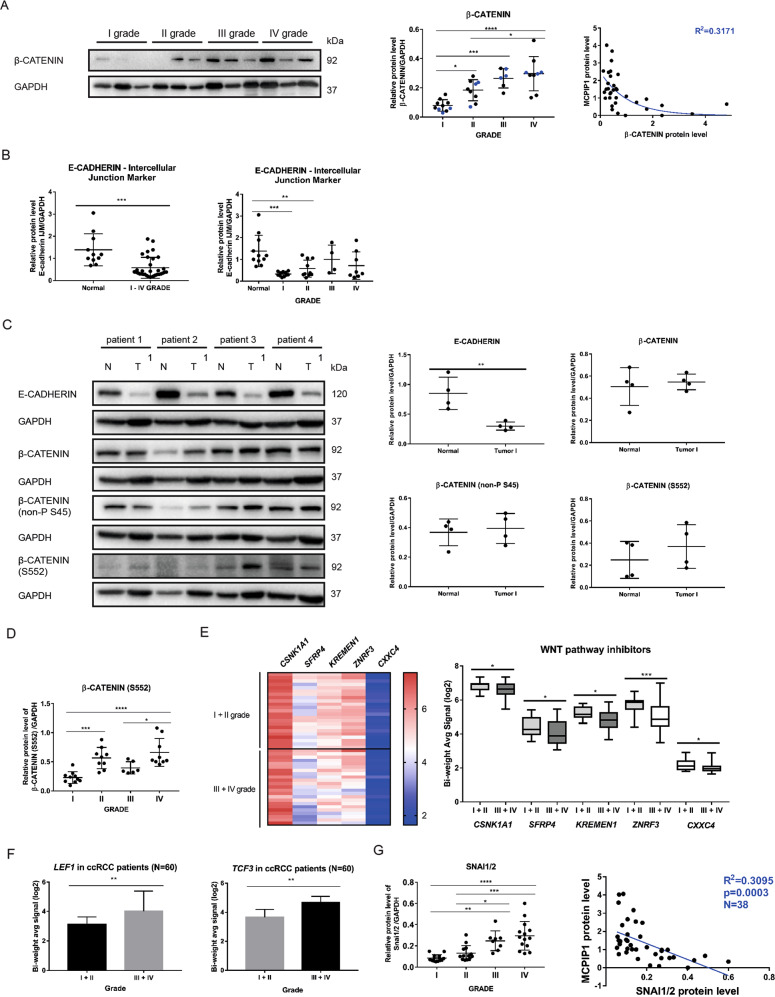
MCPIP1 inhibits the Wnt/β-catenin pathway during ccRCC progression. **A** Analysis of β-catenin levels in tumor tissue specimens from patients. Representative western blot of 12 samples with GAPDH as the loading control. Quantification of total β-catenin level in tumor samples, divided into four groups according to tumor grade (I–IV), *N* = 34. Blue samples were taken for representative western blots. Right panel, correlation of protein level between MCPIP1 and β-catenin in all patients used in the experiment, the *R*-square value is given. *P* values were estimated using one-way ANOVA. *P < 0.05, **P < 0.01, ***P < 0.001, ****P < 0.0001. **B** Quantification of E-cadherin intercellular junction marker level in normal and tumor samples, *N* = 44. Quantification of E-cadherin intercellular junction marker level in normal and tumor samples, divided into four groups according to tumor grade (I–IV) with GAPDH as the loading control. *N* = 44. *P* values were estimated using one-way ANOVA. *P < 0.05, **P < 0.01, ***P < 0.001, ****P < 0.0001. **C** Analysis of E-cadherin and β-catenin levels in ccRCC samples in comparison with non-tumor tissues. Western blot and densitometric quantification of 4 patients of E-cadherin, total β-catenin, phospho β-catenin (S552) and active (non-P S45) β-catenin with GAPDH as the loading control. *P* values were estimated using two-tailed unpaired Student’s *t* test, *P < 0.05, **P < 0.01, ***P < 0.001, ****P < 0.0001. **D** Quantification of phospho β-catenin (S552) level in patient tumor samples, divided into four groups according to tumor grade (I–IV) with GAPDH as the loading control, *N* = 34. The results are presented as means ± SD. *P* values were estimated using two-tailed unpaired Student’s *t* test, *P < 0.05, **P < 0.01, ***P < 0.001, ****P < 0.0001. **E** Hierarchical clustering of genes inhibiting the Wnt pathway: *SRFP4*, *KREMEN1*, *ZNRF3*, *CSNK1A1* and *CXXC4*. Each row represents an individual tissue sample. The scale represents gene expression levels in log_2_ scale. Quantification of the signal from microarray. N (I + II) = 23, N (III + IV) = 23. Statistics was performed using one-way ANOVA between subjects (unpaired), *P < 0.05, **P < 0.01. **F** Quantification of the *LEF1* and *TCF3* signals in a microarray analysis. The scale represents gene expression levels in log_2_ scale. *N* (I + II) = 30, *N* (III + IV) = 30. *P* values were estimated using a two-tailed, unpaired Student’s *t* test; *P < 0.05, **P < 0.01. **G** Analysis of SNAI1/2 protein level in tumor tissue specimens from patients. Quantification of total SNAI1/2 level in tumor samples, divided into four groups according to tumor grade (I–IV), *N* = 38; with GAPDH as the loading control. The results are presented as means ± SD. Correlation of protein level between MCPIP1 and SNAI1/2 in patients used in the experiment, the *R*-square and *P* value were given. *P* values were estimated using one-way ANOVA. *P < 0.05, **P < 0.01, ***P < 0.001, ****P < 0.0001.

Activated β-catenin has been found to be associated with advanced kidney cancers and lower patient survival [34]. To assess β-catenin expression during ccRCC progression, we analyzed 38 tumor samples. Western blot analysis revealed that β-catenin expression varied depending on tumor grade (Fig. 7A). The lowest level of β-catenin was observed in grade I tumors, and the level gradually increased with tumor grade up to grade IV. Moreover, the level of β-catenin was negatively correlated with the level of MCPIP1 (R2 = 0.3171) (Fig. 7A). Subsequently, we analyzed the level of E-cadherin, an intercellular junction marker, in patient tissues. The largest difference between normal and tumor tissues was observed for grades I and II, where the level of E-cadherin was the lowest (Fig. 7B). To assess the level of E-cadherin and activation of β-catenin, we evaluated 4 patients and compared normal and grade I tumor tissues. We found that in each case, the level of E-cadherin in the tumor tissue decreased drastically compared to that in the normal tissue (Fig. 7C). We observed a slight increase in the level of transcriptionally active (S552) β-catenin (Fig. 7C). Analysis of 34 patient samples showed that the level of active (S552) β-catenin significantly increased with tumor progression and was the highest in grade IV tumors (Fig. 7D). Microarray analysis of tissues from patients with ccRCC showed that the levels of Wnt pathway inhibitors decreased with tumor progression (Fig. 7E). Significant differences were observed in the levels of *SFRP4*, *KREMEN1*, *ZNFR3*, *CXXC4* and *CSNK1A1*, which were considerably lower in grade III and IV tumors than in grade I and II tumors (Fig. 7E). Microarray analysis performed on 60 patient tissues revealed a significant increase in *LEF1* and *TCF3* transcription factors (Fig. 7F). Moreover, analysis of tissues from patients with ccRCC showed that the protein level of SNAI1/2 increased during the progression of ccRCC. The lowest level of SNAI1/2 was observed in grade I tumors, and the level gradually increased with tumor grade up to grade IV. Moreover, the level of SNAI1/2 was negatively correlated with the protein level of MCPIP1 (R2 = 0.3047; *P* = 0.0002) (Fig. 7G). Using The Cancer Genome Atlas (TCGA) Kidney Clear Cell Carcinoma (KIRC) database from the Genomic Data Commons (GDC), we performed the same analysis with a significantly larger group of patients (*N* = 985), and we evaluated the correlations of the gene expression level of *CTNNB1*, which encodes β-catenin, with the gene expression levels of the transcription factors *SNAI1*, *SNAI2* and *ZEB1* (Supplementary Fig. S6B). The correlation coefficient was the highest for ZEB1 (R2 = 0.6230; *P* = 1.560e–66), and correlations were also observed for SNAI2 (R2 = 0.3820; *P* = 1.605e–22) and SNAI1 (R2 = 0.2393; *P* = 2.381e–9).

## Discussion

We previously revealed that during ccRCC progression, the level of MCPIP1 decreases. Moreover, a low level of MCPIP1 in ccRCC cells increases the levels of vimentin and β-catenin and decreases that of E-cadherin, indicating that MCPIP1 may control the acquisition of mesenchymal features [24]. In the present study, we showed that the RNase activity of MCPIP1 influences the level, localization and activity of β-catenin in ccRCC cells through regulation of the expression of negative regulators of the Wnt pathway. We also demonstrated that MCPIP1 is essential for controlling the EMT process and maintaining the epithelial phenotype. MCPIP1 is a member of the Regnase family and is composed of a PilT N-terminus-like (PIN) domain; after physical interaction with stem-loop structures in the 3′ UTR of transcripts, MCPIP1 causes mRNA destabilization followed by degradation [17, 35]. MCPIP1 participates in the endonucleolytic digestion of major proinflammatory cytokines, such as IL-6, IL-1β, IL-12b [14, 27, 36], and proangiogenic IL-8 mRNA [37], which is mediated by its enzymatic activity. A single mutation, D141N, completely abolishes the RNase activity of MCPIP1 [27, 38]. Initially, in our study, we evaluated whether the loss of RNase activity influences the mRNA expression and protein levels of EMT markers. We observed increased levels of mesenchymal markers and a decreased level of E-cadherin in cells with loss of MCPIP1 RNase function and the corresponding inverse effects in cells with MCPIP1 overexpression. This finding confirms previous observations that silencing MCPIP1 decreases E-cadherin expression and increases vimentin expression [24] and indicates that the RNase activity of MCPIP1 is crucial for maintaining the epithelial phenotype.

One hallmark of EMT is the loss of E-cadherin function, which results in the dissociation of the E-cadherin-β-catenin complex from the membrane. A reduced E-cadherin level is often connected with an increased level of free cytoplasmic β-catenin, which can result in an increased level of transcriptionally active β-catenin, which promotes tumor development and progression if it escapes cytoplasmic degradation [30, 31, 39, 40]. Our results showed that the RNase activity of MCPIP1 is responsible for the loss of E-cadherin and the translocation of β-catenin into the cell nucleus. Our observations suggest that at least in this model, MCPIP1 and its RNase activity are crucial in regulating both the level of E-cadherin and the activation and localization of β-catenin. The nuclear translocation of β-catenin was previously shown to determine the activation of Wnt-responsive genes [30, 31, 40]. On the other hand, without Wnt stimulation, β-catenin is constantly degraded by the proteasome [41, 42]. This degradation strictly depends upon β-catenin phosphorylation, which is sequentially carried out by two distinct kinases, CK1 and GSK-3. CK1-mediated phosphorylation of S45 occurs first and is required for the subsequent GSK-3-mediated phosphorylation of T41, S37, and S33 [43]. Our data suggest that MCPIP1 may regulate the process of this first step (CK1-mediated phosphorylation of S45), as the loss of RNase activity of MCPIP1 led to a significant increase in the level of the active form of β-catenin, which is not phosphorylated on S45 (i.e., non-P S45). MCPIP1 not only downregulates a set of mRNAs but also acts as a suppressor of miRNA biogenesis by cleaving the terminal loops of precursor miRNAs, thus counteracting Dicer1 activity [18, 19]. In the present study, we provide evidence that MCPIP1 degrades miRNA-519a-3p, miRNA-519b-3p, and miRNA-520c-3p, which inhibit the transcription of several genes, including *SFRP4*, *ZNRF3*, *KREMEN1*, *CXXC4* and *CSNK1A1*, which act as negative regulators of Wnt signaling and reduce the level of active β-catenin. In addition, previous studies have shown that negative regulators of β-catenin and Wnt signaling play important roles in the development and progression of RCC [5–7]. The *CSNK1A1* gene encodes CK1, a component of the β-catenin destruction complex that phosphorylates β-catenin at Ser45 [44], and we speculate that the reduction in *CSNK1A1* transcription explains the significant increase in the active form of β-catenin in cells expressing MCPIP1 with a mutation in the RNase domain. ZNRF3 inhibits Wnt/β-catenin signaling by specifically mediating the ubiquitination of the Wnt receptor FZD and coreceptor LRP6 [43]. CXXC4 is a negative regulator of Wnt/β-catenin signaling in RCC, and the CXXC4 mRNA level gradually decreases in RCC as the tumor stage and grade increases [7]. The transmembrane proteins Kremen1 and Kremen2 have high affinity for the Dkk1 receptors, blocking Wnt/β-catenin signaling [45]. Another antagonist of Wnt signaling is SFRP4, which belongs to the secreted frizzled-related protein family and is frequently silenced in ccRCC tumor tissues [46]. However, MCPIP1 appears to act on multiple levels by activating the EMT process. We have already shown that MCPIP1 overexpression reduces the level of hypoxia-inducible factors (HIFs) in ccRCC [22], which may also partially affect the activity of β catenin in ccRCC [47–49]. HIF-2α has been shown to associate with the β-catenin/TCF complex and enhance the transcriptional activity of β-catenin in ccRCC cells [49]. Another study showed that β-catenin directly interacts with HIF-1α and that the formation of the HIF-1α/β-catenin complex increases the activity of HIF-1α and consequently enhances EMT in hepatocarcinoma [50]. The observed regulation of the expression of inhibitors of the Wnt pathway and influence on HIFs may explain the observed decrease in β-catenin activity in cells with MCPIP1 overexpression and its excessive activity in the absence of MCPIP1 RNase function, which facilitates the degradation of its transcripts.

The Wnt/β-catenin pathway is one of the signaling pathways that controls EMT by regulating direct or indirect transcriptional targets of the canonical Wnt pathway. Among the targets of the Wnt pathway, transcription factors repress the expression of epithelial markers such as E-cadherin and the activation of mesenchymal genes [1]. Our study revealed that in addition to regulating the level of active β-catenin, MCPIP1 regulates the expression of the transcription factors SNAI1, SNAI2, ZEB1 and TWIST via its RNA activity, which in turn activates the EMT process.

The transcriptional activity of β-catenin results from Wnt/Wingless-dependent or Wnt/Wingless-independent signaling. The phosphorylation of β-catenin by protein kinase B (AKT) at S552 was previously demonstrated stabilize the protein, enhance its nuclear accumulation and increase its transcriptional activity [8]. Our results revealed that the RNase activity of MCPIP1 accounted for the increased level of transcriptionally active (S552) β-catenin; in contrast, MCPIP1 overexpression led to a reduced level of stabilized β-catenin (S552) in the nucleus. Moreover, the level of transcriptionally active β-catenin increased with ccRCC progression in patients. Our group previously demonstrated that overexpression of MCPIP1 resulted in reduced activation of AKT and that cells lacking RNase activity showed a significant increase in the level of AKT phosphorylation [22, 25]. Moreover, protein array analysis of ccRCC patient samples showed increased levels of phosphorylated mTOR and AKT 1/2/3 kinases, which are related to tumor invasiveness and metastasis [25]. These findings might partially explain our observations of increased levels of transcriptionally active (S552) β-catenin in ccRCC. However, β-catenin may undergo other modifications that regulate its activity, which may be dependent or independent of the activity of MCPIP1.

**Fig. 8:**
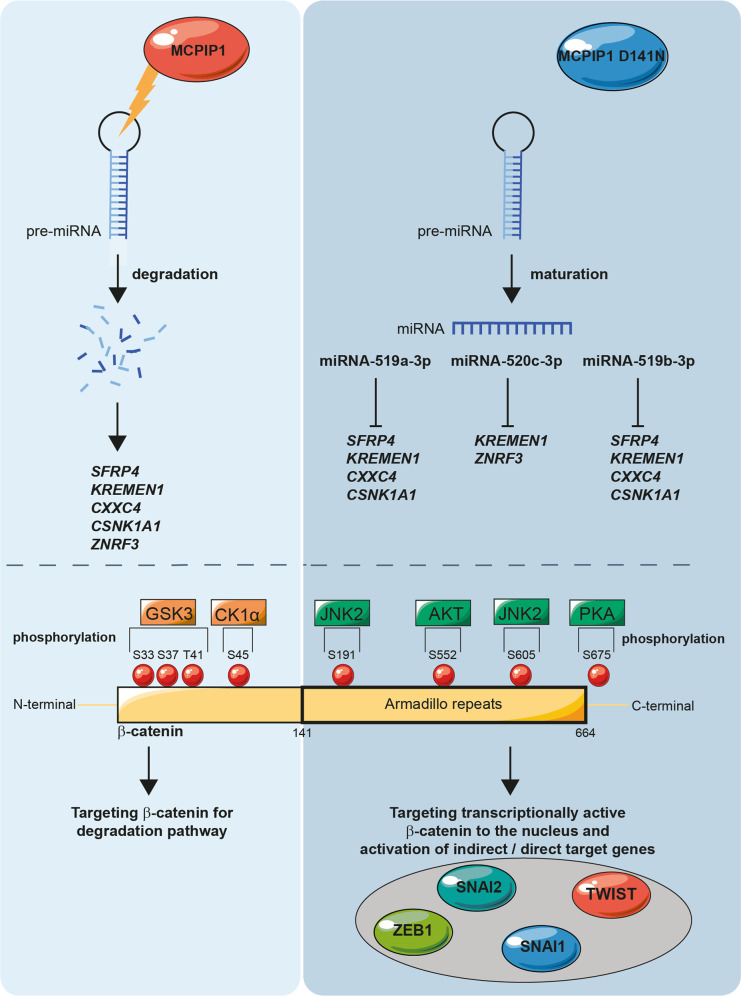
The possible mechanism by which MCPIP1 regulates the EMT process and the β-catenin level. (Left panel) MCPIP1 regulates the levels of miRNA thereby actively influencing the levels of *SFRP4*, *KREMEN1*, *ZNRF3*, *CXXC4* and *CSNK1A1* and inhibiting the Wnt pathway by inactivating β-catenin and consequently inhibiting the EMT process. (Right panel) The loss of MCPIP1 RNase activity protects miRNA-519a-3p, miRNA-519b-3p, and miRNA-520c-3p from degradation; these miRNAs mature and inhibit the expression of negative regulators of the Wnt/β-catenin pathway, thus regulating the level of active β-catenin and the EMT process.

Collectively, these observations indicate the role of MCPIP1 as a possible regulator of the EMT process that prevents cells from acquiring the mesenchymal phenotype. In conclusion, we provide evidence that the endoribonuclease activity of MCPIP1 regulates the levels of miRNA-519a-3p, miRNA-519b-3p and miRNA-520c-3p, thereby actively influencing the levels of SFRP4, KREMEN1, ZNRF3, CXXC4 and CSNK1A1 and inhibiting the Wnt pathway by inactivating β-catenin and consequently inhibiting the EMT process. Furthermore, we show that the loss of MCPIP1 RNase activity protects miRNA-519a-3p, miRNA-519b-3p, and miRNA-520c-3p from degradation; these miRNAs can then mature and inhibit the expression of negative regulators of the Wnt/β-catenin pathway, thus regulating the level of active β-catenin and the EMT process. We propose a possible mechanism by which MCPIP1 regulates the EMT process and the β-catenin level (Fig. 8).

However, the regulation of EMT by MCPIP1 and the mechanistic relationship between MCPIP1 and Wnt signaling in ccRCC are not entirely clear. There are still open questions about the role of MCPIP1 in cancer cell fate. The current knowledge about the MCPIP1 protein indicates that it regulates cell fate not only directly, thanks to its RNase function, but also indirectly, by affecting the microenvironment. By regulating cytokine and chemokine transcripts, it affects the surrounding cells, which may interact with cancer cells in response. MCPIP1 can also influence the EMT process by directly regulating IL6 expression and secretion. IL6 has already been shown to induce EMT and tumor progression [51], and we have demonstrated that MCPIP1 directly regulates the levels of IL-6 in ccRCC in vitro and in vivo [22, 24]. We also previously showed that the loss of TIMP3 in cells and in tumors generated in NOD-SCID mice lacking RNase activity led to increased IL-6 expression and was correlated with tumor progression [25].

Our study has some limitations. MCPIP1 may act as an endonuclease on the transcripts of other genes that we have not studied. The use of shRNA to knockdown MCPIP1 leaves some MCPIP1 biological activity in the event of incomplete silencing. Moreover, there are probably other functions of the MCPIP1 protein that are not related to the PIN domain function, which should be elucidated in further studies. However, our data highlight the importance of MCPIP1 as a suppressor of tumor development and progression and show that MCPIP1 is crucial for cell fate regulation as it controls the acquisition of the mesenchymal phenotype, the activation of β-catenin and the induction of target gene transcription.

## Materials and methods

### Microarray analysis and patient tissue samples

Fragments of tumor tissues from patients diagnosed with ccRCC were collected using protocols approved by the Local Ethics Committee (approval no. 68/KBL/OIL/2011) at the Center of Oncology, Maria Sklodowska-Curie Memorial Institute, Cracow Branch, Poland, with conscious consent of the patients. Samples were frozen in liquid nitrogen and stored at −80 °C for protein isolation or incubated overnight in RNAlater (Invitrogen, MA USA) at 4 °C and stored at −80 °C for RNA isolation. A detailed description of the microarray assay is provided in the Supplementary Methods.

### Cell culture

Caki-1 (ATCC, VA USA) and A549 (ATCC) cells were cultured in Eagle’s minimal essential medium (Lonza, Switzerland) supplemented with 10% FBS (Sigma, Germany). Caki-2 cells (Sigma) were cultured in McCoy’s 5A medium (BioWest, MO USA) supplemented with 10% FBS. The RPTEC/TERT1 (ATCC) cell line (passages 7–17) was cultured in DMEM:F12 medium (ATCC) supplemented with the components of the hTERT RPTEC Growth Kit (ATCC) and G418 at a final concentration of 0.1 mg/ml (Sigma).

### Viral vectors

To overexpress MCPIP1 in cell lines, the following lentiviral vectors based on the doxycycline-dependent TetON system were used: pLIX MCPIP1 (MCPIP1), control pLIX PURO (PURO) and mutant form pLIX D141N (MCPIP1-D141N). To stably inhibit MCPIP1 in the RPTEC/TERT1 cell line, shRNA (shMCPIP1) and control (shCtrl) lentiviral vectors (Sigma) were used at a multiplicity of infection (MOI) of 50.

### miRNA sequencing

For RNA sequencing, total RNA was extracted using the mirVana miRNA Isolation Kit (Ambion, MA USA) according to the manufacturer’s protocol. The concentration and purity of the isolated RNA were measured with a NanoDrop 1000 spectrophotometer, and RNA integrity was checked on a 1% denaturing agarose gel. High-throughput miRNA sequencing was conducted on the Ion Torrent Proton platform. The small RNA fraction in total RNA samples was assessed using a Small RNA Kit (Chip Kit) and an Agilent 2100 Bioanalyzer. A total of 3.5 µg of total RNA per sample was used for library preparation with the Ion Total RNA-Seq Kit v2 (Thermo Fisher Scientific, MA USA) following the manufacturer’s protocol for small RNA library preparation. Nine barcoded libraries were combined, pooled in equimolar concentrations and subsequently sequenced on a single Ion PI Chip v3 using an Ion Proton Sequencer and Ion PI Hi-Q Sequencing 200 chemistry. The raw sequencing data were preprocessed using tools available on the Torrent Suite Server v5.10.0. Subsequently, reads were mapped to the human genome (hg19), counted and subjected to differential expression analysis with the DESeq2 package (with default parameters) implemented in R version 3.3.3 software. The *P* values for differentially expressed miRNAs were corrected for multiple comparisons using the Benjamini–Hochberg approach, and miRNAs with corrected *P* values < 0.05 were considered significantly differentially expressed. Target genes of the differentially expressed miRNAs were predicted using the miRNA target prediction website Diana microT-CDS [52, 53].

### miRNA power inhibitors

To inhibit miRNA expression, miRCURY LNA miRNA Family Power Inhibitors from Qiagen (MD, USA) were used. Caki-1 cells (30,000) were seeded in a 24-well plate. After 24 h, to induce overexpression, doxycycline was added. After another 24 h, miRNA Power Inhibitors targeting miRNA-519a-3p, miRNA-519b-3p and miRNA-520c-3p were added at a total concentration of 3 µM. Negative Control A was used at a concentration of 3 µM. After 72 h, total cellular RNA was isolated using the Universal RNA Purification Kit (EURx, Poland).

### Animal studies

The in vivo experiments were carried out in accordance with the Institutional Animal Care and Use Committee: II Local Ethics Committee of the Institute of Pharmacology, Polish Academy of Sciences [approval nos. 20/2017, 21/2017 and 53/2019 (163/2020)]. Mice were handled according to the regulations of national and local animal welfare bodies. Six-week-old female Foxn1^nu^/Foxn1^nu^ (Envigo, Indianapolis, USA; or Charles River Laboratory, Wilmington, Massachusetts, USA) and NOD-SCID (Charles River Laboratory) mice were housed under specific-pathogen-free (SPF) conditions with *ad libitum* access to water and food. Resuspended Caki-1 cells (2.5×106 cells in PBS) with stable overexpression or mutation of MCPIP1 (pLIX PURO, pLIX D141N, pLIX MCPIP1) or downregulation of MCPIP1 (shCTRL, shMCPIP1) were injected into the mice subcutaneously. Tumor growth was monitored for 6 weeks. After tumor excision, samples were frozen in liquid nitrogen and stored at −80 °C. Next, RNA was isolated via the phenol-chloroform method, and real-time RT-PCR analysis was performed.

### Statistical analysis

Experiments were conducted with three independent replicates. The results are shown as the mean ± SD. Statistical significance was determined using two-tailed unpaired Student’s *t* test, one-way ANOVA or two-way ANOVA as calculated with GraphPad Prism 7. *P* values for differentially expressed miRNAs were corrected for multiple comparisons using the Benjamini–Hochberg approach (*P < 0.05, **P < 0.01, ***P < 0.001, ****P < 0.0001).

For the methods used for transduction, western blot analysis, mRNA extraction, real-time PCR analysis, miRNA validation, staining and microarray analysis, please see the “Supplementary Methods” document.

## Supplementary information

Supplementary Methods

Supplementary Figures
