# Supplementary material for: MCPIP1 inhibits Wnt/β-catenin signaling pathway activity and modulates epithelial-mesenchymal transition during clear cell renal cell carcinoma progression by targeting miRNAs
Source: Oncogene. 2021 Oct 16;40(50):6720–35. doi: 10.1038/s41388-021-02062-3 (PMC8677621; doi:10.1038/s41388-021-02062-3)
Supplement: Supplementary file 1 — Supplementary Methods [file 41388_2021_2062_MOESM1_ESM.docx]

**Supplementary methods**

**Microarray assay and patient tissue samples**

The microarray assay was performed using Affymetrix HuGene ST 2.1 microarrays on 36 tumour tissue samples isolated and evaluated by pathologist. Samples were divided according to Fuhrman scale into four groups I-IV (23 samples for I + II and 23 samples for III + IV). Total RNA isolation was performed using the Eurx Universal RNA Purification Kit (EURx) according to the manufacturer's protocol. The quantity of ribosomal RNA and DNA impurities were examined by electrophoresis in 1% denaturing formaldehyde gel. Total RNA concentration was assessed using a NanoDrop 1000 spectrophotometer (Thermo Fisher Scientific) and ss-cDNA amplification was allocated 100 ng total RNA, followed by fragmentation of the sample and biotin labeling. The whole experiment was performed according to the Affymetrix GeneChip WT PLUS Reagent Kit. Next, 10 µg cRNA was hybridized for 16 hours at 48° C on Affymetrix HuGene2.1 ST Array Strip. Arrays were washed and stained at Affymetrix Gene Atlas Fluidics Station. HuGene2.1 ST Array Strips were scanned using the GeneAtlas Imaging Station. The data were normalized with Expression Console Software 1.4.1 with RMA algorithm and analyse using Affymetrix Transcriptom Analysis Console (TAC) Software 3.1 with ANOVA one-way between subjects (unpaired). We have followed the Minimum Information About a Microarray Gene Experiment (MIAME) guideline and deposited raw and processed data in the Gene Expression Omnibus (GEO) repository with the accession number: GSE150404.

To isolate the protein, the samples were divided into smaller fragments, homogenized in protein lysis buffer, and used for western blot analysis.

**Cell culture**

To passage RPTEC/TERT1 cells, 0.25% (w / v) trypsin-0.53 mM EDTA solution (ATCC) was used, followed by 0.1% Soybean Trypsin Inhibitor (ATCC) to stop trypsinization. Cell lines were cultured at 37°C in a 5% CO_2_ atmosphere. The cell lines have been tested every 4 months for mycoplasma contamination. Cell lines were authenticated by GenMed, using STR DNA profiling methods within loci of *D10S1248, vWA, D16S539, D2S1338, AMEL D8S1179, D21S11, D18S51, D22S1045, D19S433, TH01, FGA, D2S441, D3S1358, D1S1656, D12S391*.

**Transduction**

Cells were cultured on 6-well plates to 50% confluence. Lentiviral vectors were added at multiplicity of infection (MOI) of 50 with 6ug/mL polybrene (Millipore). After 24 hours, the medium was changed and a selective antibiotic puromycin (1µg/mL; InvivoGen) was added after a further 24 hours. To induce overexpression, doxycycline was added (1µg/ml; BioShop) for 48h. TGFβ1 (Sigma) stimulation was performed at 5 ng / ml for 96h, 7 and 10 days, changing the fresh medium from TGFβ1 every 48h.

**Western blot**

High-quality nuclear and cytoplasmic fraction isolation was performed using Nuclear Extract Kit (Active Motif). The whole fraction from cells was lysed for 10 minutes on ice with Mammalian Protein Extraction Reagent (Thermo Fisher Scientific) supplemented with proteinase inhibitors: aprotinin 10 μg/ml (Sigma), leupeptin 10 μg/ml (BioShop), pepstatin 1 μg/ml (Sigma), sodium orthovanadate 1 mM (Sigma) and phosphatase inhibitors: sodium fluoride 5 mM (Sigma), sodium pyrophosphate tetrabasic 5 mM (Sigma), EDTA 2.5 mM (BioShop) and PMSF (Phenylmethanesulfonyl fluoride; Sigma) 40 μg/ml. The SDS-PAGE electrophoresis of proteins was conducted in 10% the polyacrylamide gel. Next, wet transfer of protein from the gel to a PVDF membrane (Millipore) was performed, and membranes were blocked in 5% BSA in Tris-buffered saline with 0,1% Tween20 (BioShop). Membranes were incubated overnight at 4°C with the primary antibodies. Then, a 3-fold rinsing in TBS-T buffer for 10 minutes and a 1-hour incubation in the secondary antibodies were performed at room temperature. Membranes were then incubated for 5 minutes in 1ml substrate reagent (Immobilon TM Western Chemiluminescent HRP Substrate). The ChemiDoc system (Biorad) was used to detect the signal. Phosphorylated proteins were stripped using 0.2 M NaOH and re-blocked in 5% BSA. All antibodies specifications are included in Table No.1.

**mRNA extraction and RT-PCR analysis**

Total cellular RNA was isolated using the Universal RNA Purification Kit (EURx). RNA from total mice lungs and livers were isolated using Fenozol (A&A Biotechnology). The quantity of ribosomal RNA and DNA contamination was examined using electrophoresis in 1% denaturing formaldehyde gel. RNA concentration was determined spectrophotometrically (NanoDrop; Thermo Fisher). Reverse transpription was performed using 1000ng of total RNA, M-MLV reverse transcritpase (Promega), oligo(dT) primer (1 μg/μl; Promega) and dNTP (10mM, Promega). Real-time PCR was performed using QuantStudio 3 (Thermo Fisher Scientific) with SybrGreen Master Mix (A&A Biotechnology) and specific primers (10mM; Sigma). For the examination of mice lung and liver metastasis, specific probes for human GAPDH and mouse GAPDH (Life Technologies) were used with Taq PCR Master Mix (EURx). The reaction was normalized to the expression of the elongation factor-2 (*EF2*) and carried out in duplicates. The results were analyzed with QuantStudio Software with ΔΔC_t_ method. Primer sequences are shown in Table No. 2

**Validation of miRNAs**

miRNA sequencing data were confirmed using qRT-PCR. For miRNA analysis on cell line Caki-1 and mouse xenotransplant model, RNA isolation was performed with phenozole-chloroform. Reverse transcription was done with the miRCURY LNA RT Kit (Qiagen) and 10 ng of total RNA was used. Real-time PCR was performed with the miRCURY LNA SYBR Green PCR Kit (Qiagen) and QuantStudio 3. Gene expression was normalized to small RNA (*U6*) and carried out in duplicates. Relative quantification of miRNA expression was calculated using the comparative ΔΔC_t_ method with QuantStudio Software and relative miRNA expression is presented as log_2_fold differences.

Sequence of miRNA primers are listed in Table No. 3

**Immunofluorescence staining**

Coverslip cultures were fixed in 4% paraformaldehyde, permeabilized by 1% Triton X-100 in phosphate-buffered saline (PBS) and blocked with 0.2% Triton X-100 in 1% BSA in PBS. Samples with primary antibodies were incubated at 4°C overnight and with secondary antibodies for 1 h in the dark at room temperature. Used antibodies are shown in Table No. 4. Slides were mounted with glass coverslips using 10µl mounting medium (ProLong Gold Antifade) with DAPI to visualize nuclei and kept in the fridge for several hours before sealing with nail polish. Leica DMC5400 fluorescence microscope (Leica Microsystems) with 63x oil immersion objective with Leica LASX image acquisition software were used to visualize slides.

**Confocal imaging**

Slides were prepared as described above on round coverslips with a thickness of 0.17 mm. DNA counterstaining with Hoechst (Thermo Fisher Scientific). Each sample was mounted in a metal holder that closes sample in a chamber to provide best conditions during imaging, which was carried out with Leica TCS SP5 II microscope (Leica Microsystems, Germany) equipped with 63x objective (oil immersion and numerical aperture 1.4). Fluorescence of Alexa Fluor 488 was excited by 488 nm line of an argon laser and registered by a photomultiplier that collected light in a range 500 – 600 nm. Settings for Hoechst were: 405 nm light for excitation and a range of registered emission 425 – 470 nm. A confocal pinhole was set to 1 Airy.

**IHC staining**

Immunohistochemical evaluation for tumours sections was performed using primary β-catenin—1:50 (BD Biosciences, Franklin Lakes, NJ, USA), primary Non-phospho (Active) β-catenin—1:1000 (Cell Signaling) and EnVision Detection Systems Peroxidase/DAB, Rabbit/Mouse (Dako, Glostrup, Denmark). The images were taken using a Leica DMC5400 fluorescence microscope with a 10× and 20× objective with the Leica LAS X image acquisition software.

**PCR Array analysis**

The RT² Profiler™ PCR Array Human Wnt Signaling Pathway (Qiagen) that could simultaneously detect genes related to the Wnt signaling pathway was used. Total RNA was isolated using the Universal RNA Purification Kit (EURx). RT^2^ First Strand Kit (Qiagen) was obtained for cDNA synthesis and elimination of genomic DNA in RNA samples. The real-time PCR was performed with QuantStudio 3 according to the RT^2^ Profiler PCR Array instructions. Each array contained four separate housekeeping genes (*18S, GAPDH, HPRT1, GUSB*) that were used for normalization of the sample data. The results are presented as one experiment. Microarray data was normalized against the house keeping genes with QuantStudio Software and using ΔΔC_t_ for each gene of interest in the plate.

Table no.1.

List of antibodies with dilutions.

| **Antibody** | **Dilution** | **Producer** |
| --- | --- | --- |
| Rabbit anti-MCPIP1 | 1:1000 | GenTex 110807 |
| Mouse anti-α-tubulin | 1:1000 | Calbiochem CP06 |
| Mouse anti-GAPDH | 1:1000 | Cell Signaling 5174 |
| Mouse anti-β-actin | 1:1000 | Sigma 1978 |
| Rabbit anti-β-catenin | 1:1000 | BD Biosciences 610154 |
| Rabbit anti-Phospho-β-catenin (S552) | 1:1000 | Cell Signaling 9566 |
| Rabbit anti-Non-phospho (Active) β-Catenin | 1:1000 | Cell Signaling 19807 |
| Mouse anti-E-cadherin | 1:1000 | BD Biosciences 610181 |
| Rabbit anti-E-cadherin | 1:10000 | Abcam 40772 |
| Rabbit anti-N-cadherin | 1:1000 | Abcam 18203 |
| Rabbit anti-Vimentin | 1:1000 | Cell Signaling 5741 |
| Rabbit anti-Snai1 | 1:1000 | Cell Signaling 3879 |
| Rabbit anti-Snai2 | 1:1000 | Cell Signaling 9585 |
| Rabbit anti-Fibronectin | 1:1000 | Abcam 23750 |
| Mouse anti-TATA binding protein | 1:1000 | Abcam 51841 |
| Rabbit anti-SNAIL + SLUG | 1:2000 | Abcam 180714 |
| Goat anti-rabbit IgG-HRP | 1:4000 | Santa Cruz Biotechnology 2375 |
| Goat anti-mouse IgG-HRP | 1:4000 | Santa Cruz Biotechnology 516102 |

Table No. 2

Primers used in the study. Sequences are written as 5’ 🡪 3’.

| **Gene name** | **Primers** | **Accession number** |
| --- | --- | --- |
| ***ZC3H12A (MCPIP1)*** | GGAAGCAGCCGTGTCCCTATG | NM_001323551.2 |
|  | TCCAGGCTGCACTGCTCACTC |  |
| ***EF2*** | GACATCACCAAGGGTGTGCAG | NM_001961.4 |
|  | TCAGCACACTGGCATAGAGGC |  |
| ***β-CATENIN*** | AAAATGGCAGTGCGTTTAG | NM_001330729.2 |
|  | TTTGAAGGCAGTCTGTCGTA |  |
| ***E-CADHERIN*** | GAAGGTGACAGAGCCTCTGGAT | NM_001317185.2 |
|  | GATCGGTTACCGTGATCAAAATC |  |
| ***N-CADHERIN*** | GCCCCTCAAGTGTTACCTCAA | NM_001308176.2 |
|  | AGCCGAGTGATGGTCCAATTT |  |
| ***VIMENTIN*** | TCTACGAGGAGGAGATGCGG | NM_003380.5 |
|  | GGTCAAGACGTGCCAGAGAC |  |
| ***SNAI2*** | TGTTGCAGTGAGGGCAAGAA | NM_003068.5 |
|  | GACCCTGGTTGCTTCAAGGA |  |
| ***SFRP4*** | GTTGACTGTAAACGCCTAAG | NM_003014.4 |
|  | GTTTTTGCTGAGATACGTTG |  |
| ***KREMEN1*** | GATTATAGGGGAACACAGAAC | NM_001039570.3 |
|  | TTTCTGCAATAGTTGTGCTC |  |
| ***ZNRF3*** | ACAACCCACTGAATACTTTG | NM_001206998.2 |
|  | TCAGCTTGATTTTGACAAGG |  |
| ***CSNK1A1*** | GTTTTATGTAAGGGGTTTCCTG | NM_001271741.2 |
|  | CATATTGATGGTTCAGGGTC |  |
| ***CXXC4*** | TTCACTAGAGAGAACACCTG | NM_025212.4 |
|  | TTCTTCAGTGGTGGACTAAG |  |
| ***ZEB1*** | AAAGATGATGAATGCGAGTC | NM_001323654.1 |
|  | TCCATTTTCATCATGACCAC |  |
| ***SNAI1*** | CTCTAATCCAGAGTTTACCTTC | NM_005985.3 |
|  | GACAGAGTCCCAGATGAG |  |
| ***TWIST*** | GTCCGCAGTCTTACGAGGAG | NM_000474.3 |
|  | TGGAGGACCTGGTAGAGGAA |  |
| ***LEF1*** | AGAGAGAGAAACTACAGGAATC | NM_001130713.3 |
|  | CCACCATGTTTCAGATGTAG |  |
| ***TCF3*** | AGATCCTTGGAAGAAAGTGG | NM_031283.3 |
|  | TTCTTACCATAGTTGTCCCG |  |

Table No. 3

Sequences are written as 5’ 🡪 3’.

| **Gene name** | **Primers (Qiagen)** |
| --- | --- |
| ***U6*** | CGCAAGGATGACACGCAAATTC |
| ***miRNA-519a-3p*** | AAAGUGCAUCCUUUUAGAGUGU |
| ***miRNA-519b-3p*** | AAAGUGCAUCCUUUUAGAGGUU |
| ***miRNA-520c-3p*** | AAAGUGCUUCCUUUUAGAGGGU |

Table No. 4

List of antibodies with concentration.

| **Antibody** | **Concentration** | **Producer** |
| --- | --- | --- |
| Rabbit anti-β-catenin | 1:50 | BD Biosciences 610154 |
| Rabbit anti-Phospho-β-catenin (S552) | 1:250 | Cell Signaling 9566 |
| Rabbit anti-Non-phospho (Active) β-Catenin | 1:800 | Cell Signaling 19807 |
| Rabbit anti-Fibronectin | 1:250 | Abcam 23750 |
| Goat anti-rabbit AlexaFluor 488 | 1:1000 | Thermo Fisher Scientific A-11008 |
| Goat anti-mouse AlexaFluor 546 | 1:1000 | Thermo Fisher Scientific A-11030 |
| Hoechst | 1µg/ml | Thermo Fisher Scientific H3569 |
