# Supplementary material for: MCPIP1 inhibits Wnt/β-catenin signaling pathway activity and modulates epithelial-mesenchymal transition during clear cell renal cell carcinoma progression by targeting miRNAs
Source: Oncogene. 2021 Oct 16;40(50):6720–35. doi: 10.1038/s41388-021-02062-3 (PMC8677621; doi:10.1038/s41388-021-02062-3)
Supplement: Supplementary file 2 — Supplementary Figures [file 41388_2021_2062_MOESM2_ESM.pdf]

SUPPLEMENT 1

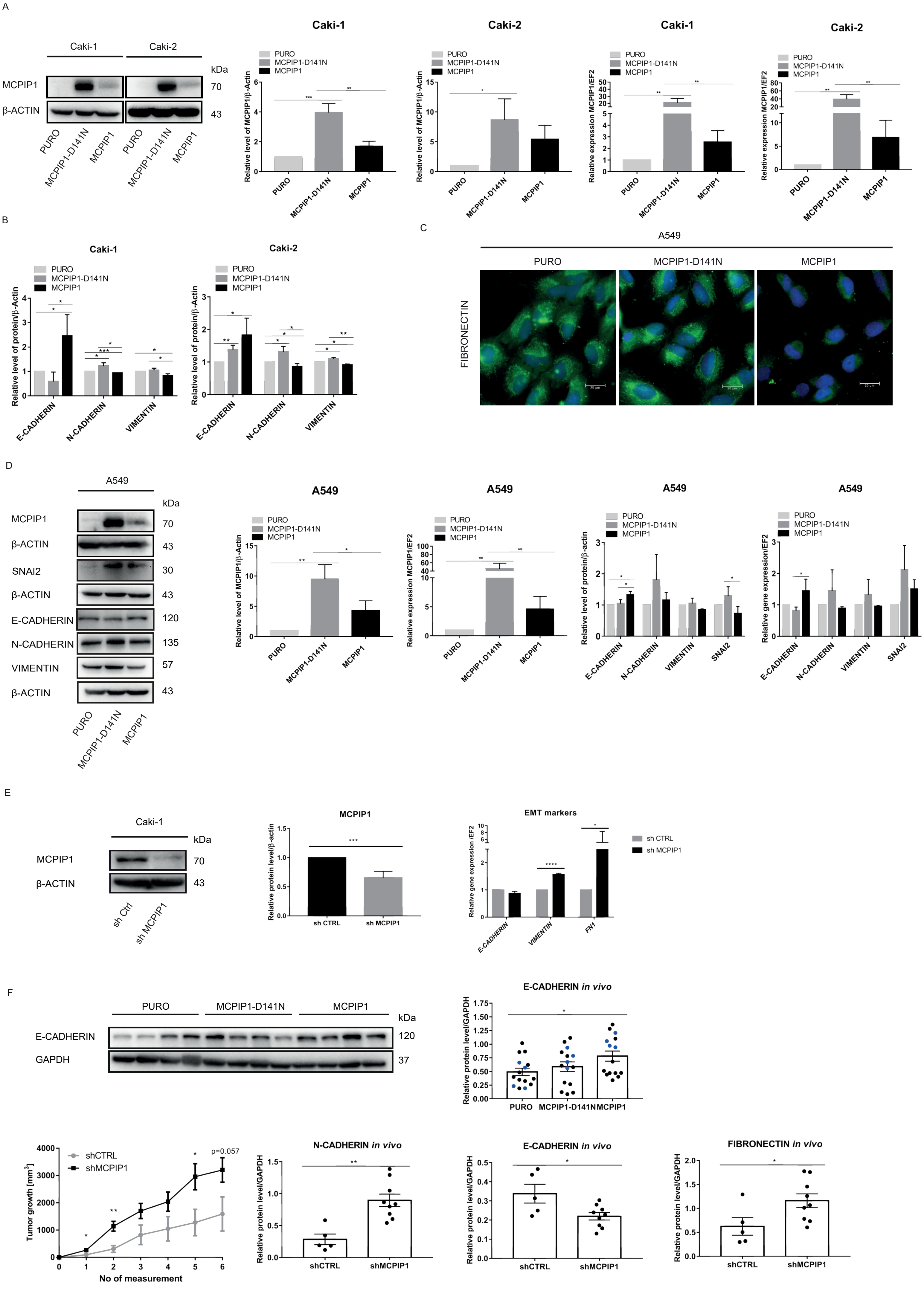

Supplementary Figure 1

(A) Representative western blot of MCPIP1 in ccRCC cell lines after overexpression of MCPIP1, MCPIP1-D141N and PURO (left panel) with β-actin as a loading control. Densitometric quantification (middle panel), PURO was set to 1. mRNA expression of MCPIP1 (right panel) in ccRCC cell lines after overexpression of MCPIP1, MCPIP1-D141N and PURO, *EF2* was used as the reference gene. The results are presented as the mean±SD of three independent experiments. *P*-values were estimated using one-way ANOVA, $*P < 0.05$, $**P < 0.01$, $***P < 0.001$.

(B) Densitometric quantification for EMT markers for Caki-1 and Caki-2 cell lines, PURO was set to 1. The results are presented as the mean±SD of three independent experiments. *P*-values were estimated using one-way ANOVA, $*P < 0.05$, $**P < 0.01$, $***P < 0.001$.

(C) Immunofluorescence staining on fibronectin of A549 cells after overexpression of MCPIP1, MCPIP1-D141N, and PURO. DAPI for nuclei; fibronectin antibody labeled with fluorescent dye AlexaFluor 546, scale bar = 20 µm.

(D) Representative western blot of MCPIP1 and EMT markers in A549 cells: SNAI2, E-cadherin, N-cadherin, and Vimentin after overexpression of MCPIP1, MCPIP1-D141N and PURO with β-actin as a loading control. Densitometric quantification, PURO was set to 1. mRNA expression of MCPIP1 in A549 cell line after overexpression of MCPIP1, MCPIP1-D141N and PURO, *EF2* was used as the reference gene. Densitometric quantification for EMT markers for A549 cell line, PURO was set to 1. mRNA expression of EMT markers in A549 cell line after overexpression of MCPIP1, MCPIP1-D141N and PURO, *EF2* was used as the reference gene. The results are presented as the mean±SD of three independent experiments. *P*-values were estimated using one-way ANOVA, $*P < 0.05$.

(E) Representative western blot of MCPIP1 of Caki-1 cell lines after downregulation of MCPIP1 (shMCPIP1) and control cells (shCtrl) with β-actin as a loading control. Densitometric quantification (middle panel), shCtrl was set to 1. mRNA levels of EMT markers in Caki-1, quantified with real-time PCR, *EF2* was used as the reference gene. The results are presented as the mean±SD of three

independent experiments. *P*-values were estimated using two-tailed unpaired Student's *t*-test, $*P < 0.05$, $**P < 0.01$, $***P < 0.001$.

(F) Representative western blot analysis of E-cadherin in tumors with overexpression, mutation of MCPIP1 and control (PURO) with densitometric quantification of protein levels in tumors. Densitometric quantification of E-cadherin intercellular junction marker in tumors with overexpression, mutation of MCPIP1 and control (PURO). Animal studies involved 45 NOD-SCID mice: PURO N=15, MCPIP1-D141N N=15, MCPIP1 N=15; GAPDH as a loading control. Effect of MCPIP1 downregulation (shMCPIP1) on tumor growth *in vivo*. Caliper measurements of tumor growth for 6 weeks. Effect of MCPIP1 downregulation on EMT markers in xenotransplantation model in mice. Densitometric quantification of N-cadherin, E-cadherin, and fibronectin in tumors with downregulation and control (shMCPIP1). The results are presented as means±SD. Animal studies involved 14 $Foxn1^{nu}/Foxn1^{nu}$ mice: shCTRL N=5, shMCPIP1 N=9. The results are presented as mean±SD. *P*-values were estimated using one-way ANOVA and two-tailed unpaired Student's *t*-test, $*P < 0.05$, $**P < 0.01$, $***P < 0.001$.

SUPPLEMENT 2

A

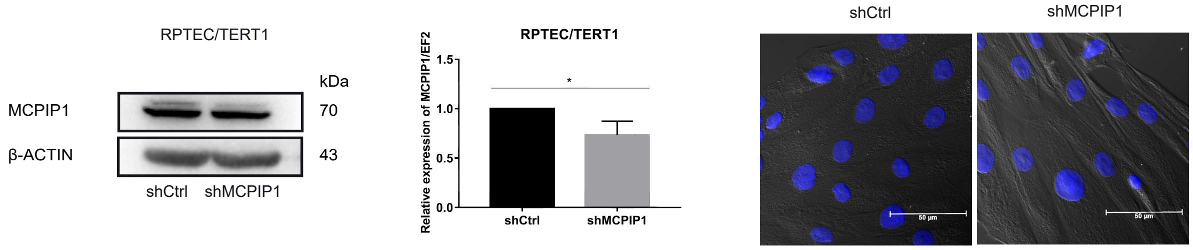

B

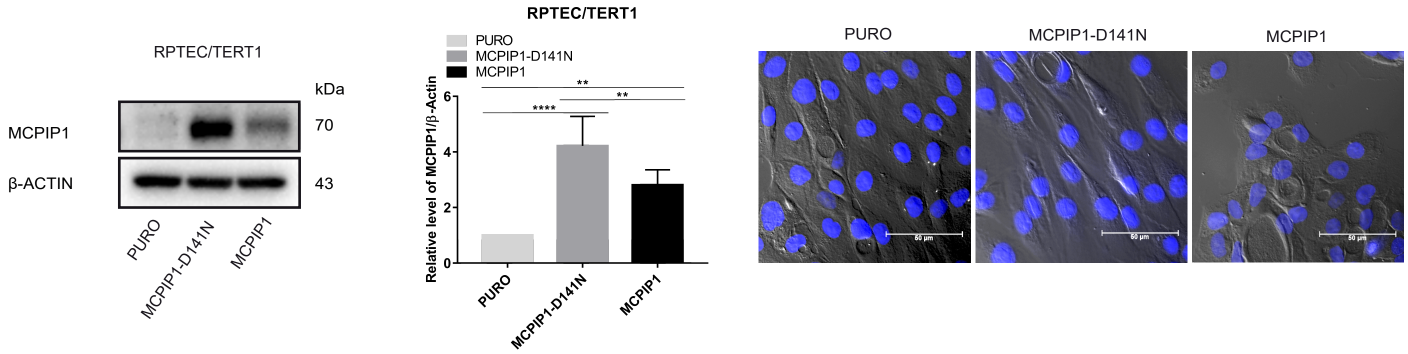

C

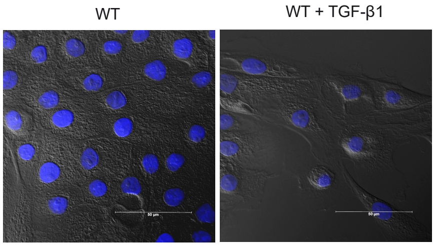

Supplementary Figure 2

(A) Morphology of normal epithelial cell line RPTEC/TERT1 after downregulation of MCPIP1 (shMCPIP1). DAPI for nuclei; scale bar = 50 μm. Representative western blot of MCPIP1 in RPTEC/TERT1 cell line after downregulation of MCPIP1 with β-actin as a loading control. mRNA expression of MCPIP1 in RPTEC/TERT1 cell line after downregulation of MCPIP1, *EF2* was used as the reference gene. The results are presented as the mean±SD of three independent experiments. *P*-values were estimated using one-way ANOVA and two-tailed unpaired Student's *t*-test, *$P < 0.05$, **$P < 0.01$, ***$P < 0.001$, ****$P < 0.0001$.

(B) Morphology of RPTEC/TERT1 after overexpression of MCPIP1, MCPIP1-D141N and PURO. DAPI for nuclei; scale bar = 50 μm. Representative western blot of MCPIP1 in RPTEC/TERT1 cell line after overexpression of MCPIP1, MCPIP1-D141N and PURO with β-actin as a loading control. Densitometric quantification, PURO was set to 1. The results are presented as the mean±SD of three independent experiments. *P*-values were estimated using one-way ANOVA and two-tailed unpaired Student's *t*-test, *$P < 0.05$, **$P < 0.01$, ***$P < 0.001$, ****$P < 0.0001$.

(C) Morphology of RPTEC/TERT1 wild type cells stimulated with TGF-β1. DAPI for nuclei; scale bar = 50 μm.

SUPPLEMENT 3

A

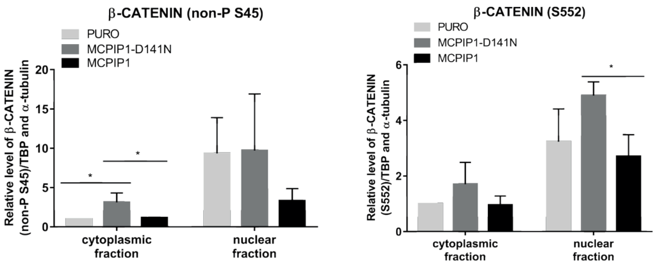

Supplementary Figure 3

(A) Densitometric quantification of β-catenin (S552) and active β-catenin, PURO in the cytoplasmic fraction was set to 1. The results are presented as the mean±SD of three independent experiments. *P*-values were estimated using one-way ANOVA, $*P < 0.05$.

SUPPLEMENT 4

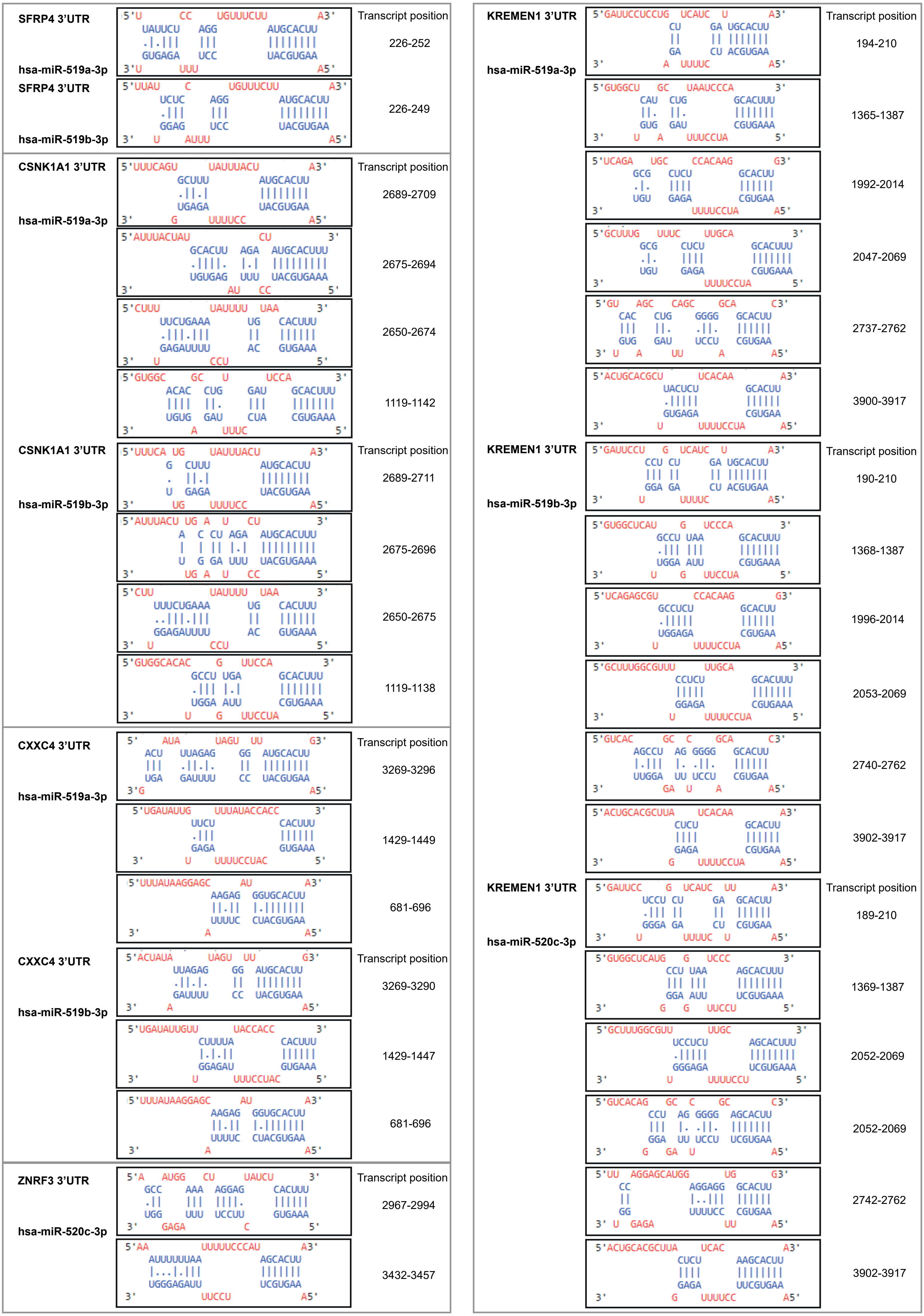

Supplementary Figure 4

Based on DIANA database, the binding sequences of the 3'UTR target genes and the tested miRNAs were presented. The data shows the sequence given and the transcript binding site of SFRP4, CSNK1A1, CXXC4, ZNRF3 and KREMEN1.

SUPPLEMENT 5
A

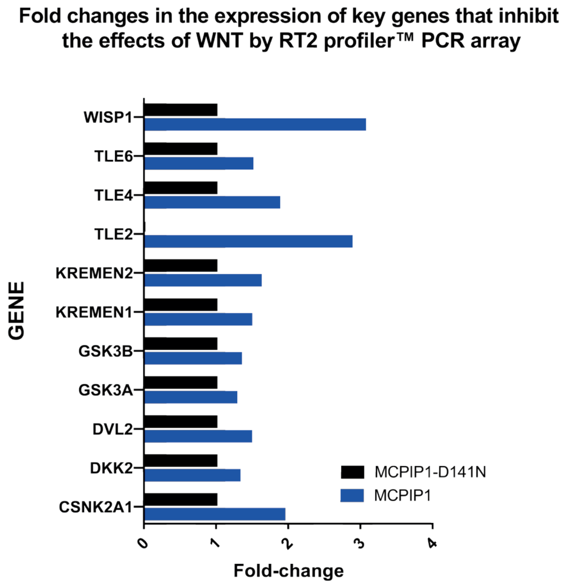

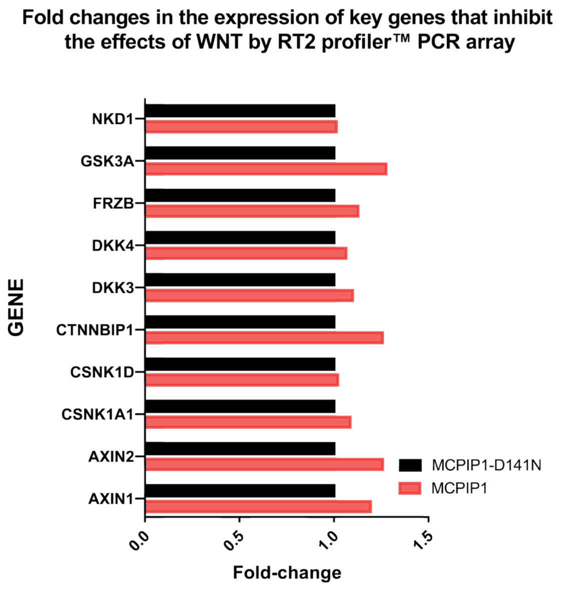

Supplementary Figure 5

(A) Fold change analysis of the mRNA level of genes inhibiting the Wnt pathway was performed on the Caki-1 line between overexpression of MCPIP1 and MCPIP1-D141N. Four reference genes *18S, GAPDH, HPRT1, GUSB* were used for normalization. The graph on the left represents fold-change greater than 1.5, the graph on the right represents fold-change less than 1.5.

SUPPLEMENT 6

A

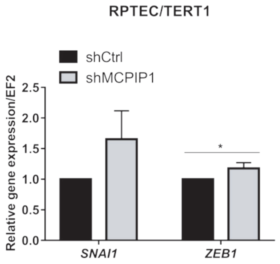

B

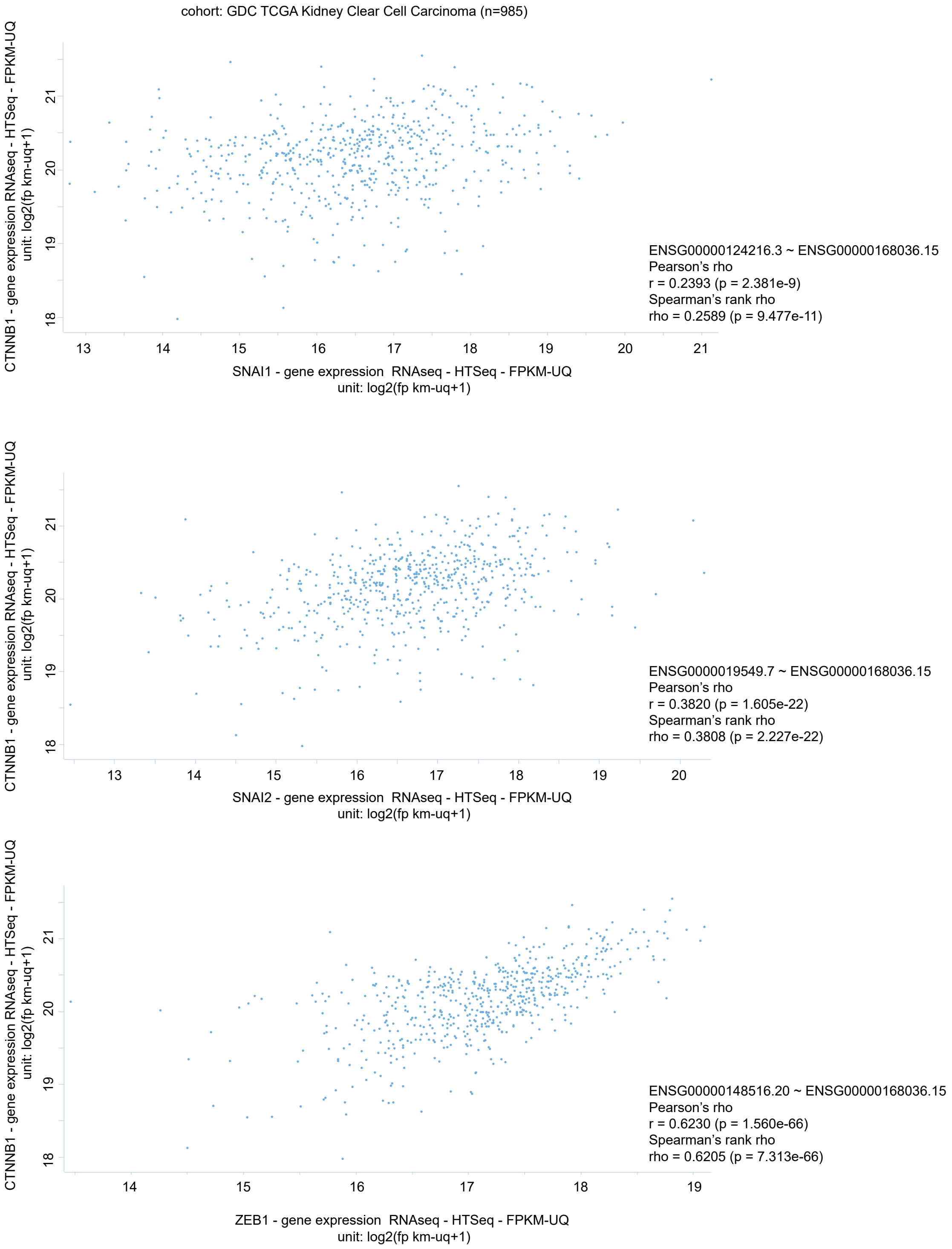

Supplementary Figure 6

(A) Effect of MCPIP1 downregulation in RPTEC/TERT1 cells in the level of *SNAI1* and *ZEB1* transcript. shCTRL was set to 1. The results are presented as the mean±SD of three independent experiments. *P*-values were estimated using two-tailed unpaired Student's *t*-test, $^*P < 0.05$.

(B) Graph showing a correlation analysis of the expression level of the *CTNNB1* gene encoding β-catenin to *SNAI1*, *SNAI2* and *ZEB1* based on the TCGA Kidney Clear Cell Carcinoma (KIRC) GDC; N = 985, R-square and P-value is given; unit: $\log_2$ (fpkm-uq + 1).
